# Supplementary material for: A Place-Based Conceptual Model (PBCM) of Neotricula aperta/Schistosoma mekongi habitat before and after dam construction in the Lower Mekong River
Source: PLoS Negl Trop Dis. 2023 Oct 6;17(10):e0011122. doi: 10.1371/journal.pntd.0011122 (PMC10584140; doi:10.1371/journal.pntd.0011122)
Supplement: Attachment — Submitted filename: PNTD-D-23-00131 (1)jrs.pdf [file pntd.0011122.s002.pdf]

PLOS Neglected Tropical Diseases

A Place-Based Conceptual Model (PBCM) of Neotricula aperta Habitat During Transmission of Schistosoma mekongi on Khong Island, Laos.

--Manuscript Draft--

**Manuscript Number:**	PNTD-D-23-00131
**Full Title:**	A Place-Based Conceptual Model (PBCM) of Neotricula aperta Habitat During Transmission of Schistosoma mekongi on Khong Island, Laos.
**Short Title:**	A PBCM of Neotricula aperta habitat
**Article Type:**	Research Article
**Keywords:**	Keywords<br><br>Neotricula aperta<br>Schistosoma mekongi<br>Vector snail habitat model<br>Place-Based Conceptual Model (PBCM)<br>E-flow habitat management<br>Lancang- Mekong River<br>Climate change dams disease
**Abstract:**	Abstract<br>A brief history with an update describing Schistosoma mekongi and its vector snail Neotricula aperta is provided. In 1971, scientists from Mahidol University in Thailand and the Smithsonian Institution in the USA formed a research team to study a new species of Schistosoma in the Mekong River in Thailand and Laos. The studies were completed during 1971-1973 prior to the construction of any dams or restrictions to the natural flow regime of the Mekong River, and provide a unique description of the natural ecological state of the river that can serve as a baseline for current research. The natural transmission of Schistosoma japonicum, Mekong Strain was first reported on Khong Island, Laos in 1973 using sentinel mice. The first detailed description of the habitat ecology of the snail vector Neotricula aperta was done on-site in 1971 simultaneous with that research and is unique in providing the only description of the river shoreline habitat before any dams were built and any alteration of the natural flow regime was in place. Aggregating current information in a Place-Based Conceptual Model (PBCM) as an organizing template along with current habitat models that combine ecological data with e-flows can be developed and used as a tool to predict suitable habitat for snails including disease vectors. We provide the original description of N. aperta habitat in Mekong River during transmission of S. mekongi. The natural flow regime of the Mekong River prior to any impoundments is described with current updates on the potential impacts of climate change and flow related snail habitat characteristics including sediment drift and water quality. Environmental management strategies are outlined emphasizing environmental flow (e-flow) and habitat models with potential to mitigate N. aperta distribution and S. mekongi disease transmission.
**Additional Information:**	
**Question**	**Response**
**Government Employee**<br><br><br>Are you or any of the contributing authors an employee of the United States government?<br><br><br>Manuscripts authored by one or more US Government employees are not	No - No authors are employees of the U.S. government.

Powered by Editorial Manager® and ProduXion Manager® from Aries Systems Corporation

copyrighted, but are licensed under a CC0 Public Domain Dedication, which allows unlimited distribution and reuse of the article for any lawful purpose. This is a legal requirement for US Government employees.<br><br>This will be typeset if the manuscript is accepted for publication.	
**Financial Disclosure**<br><br>Enter a financial disclosure statement that describes the sources of funding for the work included in this submission and the role the funder(s) played. This includes grants and any commercial funding of the work or authors.<br><br>This statement will be typeset if the manuscript is accepted for publication.<br><br>Review the submission guidelines and the instructions link below for detailed requirements and guidance.	No funding supported the research.
**Competing Interests**<br><br>On behalf of all authors, disclose any competing interests that could be perceived to bias this work.<br><br>This statement will be typeset if the manuscript is accepted for publication.<br><br>Review the instructions link below and PLOS NTDs' competing interests policy to determine what information must be disclosed at submission.	The authors have declared that no competing interests exist.
**Data Availability**<br><br>Provide a **Data Availability Statement** in the box below. This statement should detail where the data used in this submission can be accessed. This	All relevant data are within the manuscript and its Supporting Information files.

statement will be typeset if the manuscript is accepted for publication.<br><br>Before publication, authors are required to make all data underlying their findings fully available, without restriction. Review our PLOS Data Policy page for detailed information on this policy. Instructions for writing your Data Availability statement can be accessed via the Instructions link below.	

Manuscript Click here to access/download;Manuscript;28docx.docx

A place-based conceptual model (PBCM) of *Neotricula aperta* habitat during transmission of *Schistosoma mekongi* on Khong Island, Laos.

Guy R Lanza[1]

[1]Corresponding Author - Division of Environmental Sciences, College of Environmental Sciences and Forestry (ESF), State University of New York, Syracuse, NY USA

https://orcid.org/0000-0002-6606-5128

glanza40@gmail.com

E. Suchart Upatham[2]

[2]Department of Biology, Faculty of Science, Mahidol University, Rama 6 Road, Bangkok, Thailand

edupatham@gmail.com

Ang Chen[3]

[3]China Three Gorges Corporation, Wuhan, 430010 China

chenang@foxmail.com

Introduction

The first description of the habitat ~~ecology~~ supporting *Schistosoma mekongi* transmission by *Neotricula aperta (Tricula aperta)* in Laos was provided in Ecology and Resource Management in the Tropics (K.C. Misra ed., I.S.T.E Press, 1981, unpublished (S1). Some information including a map and a description of the study areas was reported in (S1). The studies were completed

during 1971-73 on Khong Island, Laos before any dams were built on the Mekong River and provide a unique baseline pre-impoundment description of the river ecology and water quality. This report provides a Place-Based Conceptual Model (PBCM) o[illegible] *N. [illegible]perta* habitat during the transmission of *S. mekongi*. The PBCM combines an update and reanalysis of historical data with new information in the context of climate change and altered river flows from dams. It includes suggestions on developing practical environmental flow (e-flow) strategies coupled with ~~ecological niche and~~ habitat suitability models that can influence the shoreline habitat along the Mekong River and potentially help in controlling disease transmission. The new PBCM model can help locate potential shoreline sites along the Mekong River that could provide habitat for snail disease ~~vectors~~ including *N. aperta.*

Brief description of the study area

The Mekong River continuum is approximately 4,909 k[illegible]eters long with an average annual discharge of 14,500 m^3/s [2]. In 1971, scientists from Mahidol University in Thailand and the Smithsonian Institution in the USA formed a research team to study a suspected new species of Schist[illegible]a in the Mekong River in Thailand and Laos. The studies provided the first description of the ecology of *N. aperta* during the transmission of *S. mekongi* in Thailand and Laos and included data on the snail community and water and sediment quality. The natural transmission of Schist[illegible]a japonicum, Mekong Strain was first reported in Laos on Khong Island (Latitude $14^0 7^/ 30^{//}$N, Longitude $105^0 51^/ 45^{//}$E) in 1973 using sentinel mice [3]. *N. [illegible]perta* has also been reported as a host for *Paragonimus veocularis* and experimentally as a host for *P. heterotremus* [4].

Shimada [5] described the high susceptibility of *N. aperta* gamma strain to *S. mekongi* from Khong Island, Laos. A 2017 survey from Khong Island noted the presence of more than 5500 *N. aperta* snails from the same shoreline areas studied in 1972 [S1] with some snails testing positive for S. mekongi [6]. More recently, Kumagai [7] developed a loop-mediated isothermal amplification (LAMP) assay for *S. mekongi* and evaluated the utility of the assay for detecting *S. mekongi* DNA in human stool and snail samples in endemic areas on Khon Island, Laos. The LAMP assay results were used to develop a risk map for monitoring *S. mekongi* and preventing epidemics. Although these surveys [6] [7] did not include the site habitat characteristics, the results strongly suggest that some of the favorable *N. aperta/S mekongi* ~~habitat conditions~~ described in the baseline pre-impoundment descriptions remain in place at Khong Island. This historical information is found in (Fig. 1-5) and (Tables 1 and 2).

Fig 1 Water quality measurements in the Mekong River during one monsoon cycle in Thailand and Laos 1972-73.

Fig. 2. Water ion analyses in the Mekong River during one monsoon cycle in Thailand and Laos 1972-73.

Fig. 3. Shoreline and offshore diel cycle water quality analyses during *S. mekongi* transmission by *N. aperta* 1972.

Fig. 4. Study site sediment analyses 1972 MeB = Mekong River at Ban Dan, Thailand; MeK = Mekong River at Khong Island, Laos.

Fig. 5. Food web diagram of Khong Island transmission areas, 1971-73.

Table 1. Algal – snail associations, Mekong River at Khong Island, Laos 1971-73.

Table 2. Distribution patterns of littoral zone snail species at Khong Island, Laos 1972

Materials and methods

The PBCM integrates information on the ecological significance of the flood pulse and the natural flow regime [8][9] with changes from dams and other barriers that disrupt the natural flow and river pulses and dramatically change the dynamics of water quality [10] and sediment distribution [11]. Most of the fragmentation and disconnection caused by the world's 2.8 million dams is due to structures associated with reservoir areas greater than 1000 m^2 [12]. The PBCM was built using methods by Brierley and Fryirs [13] and Sharma [14]. Specific methods used in the 1972 Khong Island studies are provided in (S1) and the structure and major components of the PBCM are outlined in (Fig. 6). and described as (a) *N. aperta* pre-impoundment habitat and (b) updated *N. aperta* habitat.

Fig 6 The structure and major components of a PLACE-BASED CONCEPTUAL MODEL (PBCM) of *N. aperta.*

N. aperta habitat pre-impoundment baseline descriptions 1971-73

The importance of water flow on the infection rate of *S. mekongi* was highly evident during pre-impoundment baseline studies (S1). Although specific measurement of flow was not part of these studies, shoreline areas with relatively lower flow and frequent contact by humans and animals at Khong Island Laos were associated with higher infection rates of *S. mekongi.* Offshore areas in deeper water with moving currents relatively free of human contact and the

mixing, dilution, and lack of substrate for attachment of *N. aperta* may explain the absence of significant infection (Fig 3, 5); (Table 1). Another study in the Mekong River in Thailand and Laos described the association of different water flow patterns on the bionomics of hydrobiid snails including the alpha- and gamma-strains *N. aperta*. That study reported notably higher densities of snails in areas with higher water flow, but data was not collected on the transmission of *S. mekongi* [15]. Site studies of favorable *N. aperta* ~~habitat~~ at Khong Island prior to any changes in the natural flow regime of the Lancang-Mekong River basin indicate that the site reflected similar downstream water quality and sedimentation characteristics when compared to an upstream reference site at the village of Ban Dan, Thailand (Fig 2- 4). [S1].

Water quality data measuring ten parameters from one monsoon cycle in the Mekong River prior to impoundment indicated a cyclic but stable habitat for snails, algae, and aquatic plants (Fig 1). Sediment data indicated lower levels of some parameters due to particles settling out with distance (Fig 4). Other data from downstream stations in Vietnam show similar trends in sediment transport and characteristics [16]. Statistically significant differences were noted in very fine sand, organic carbon, active iron, and Ca^{2+} (Fig. 4). Studies of substrate favored by *N. aperta* indicate that they use hard substrates such as tree branches, leaves, metal, rocks, and aquatic plants and that sand was seldom used by *N. aperta* (Fig 5; Tables 1 and 2). Sediments are also a major source of nutrients supporting the primary producer community that provides substrate and food for *N. aperta* and other snail species of the Lancang-Mekong River Basin. The microbial primary producer community on Khong Island had sixteen species that serve as

food and substrate for *N. aperta* (Fig. 5; Table 1.) and twenty-three species of dominant snails collected there (Table 2).

Updated *N. aperta* habitat descriptions.

Climate change can lead to new habitat structures for snail vec[illegible] by driving changes in weather patterns, flow regimes, water quality, sediment transfer, and other key environmental factors. Wang [17] notes that the Lower Mekong River Basin (LMRB) faces two major challenges to its water resources in the 21st century: potential climate change and ongoing and planned dam constructions. Alla [18] reviewed the impacts of dams and noted that they result in major negative impacts on water quality, aquatic ecology, land, terrestrial wildlife, vegetation, and air quality and describe and recommend mitigation measures that should be put in place to reduce these negative impacts on the environment. A changing climate could modify rainfall patterns leading to changes in the hydrological cycle, including shifts in Monsoon patterns. One report indicates that within the coming 20–30 years the operation of planned hydropower reservoirs is likely to have a larger impact on the Mekong hydrograph than the impacts of climate change, particularly during the dry season. It also predicts that climate change will increase the uncertainty of the estimated reservoir operation impacts and that the direction of the flow-related changes induced by climate change is partly unclear [19]. When all the planned dam constructions are complete, the total active capacity of the LMRB is estimated to increase from 5 km^3 to more than 100 km^3. Laos lies almost entirely within the LMRB and its climate, landscape, and land use are the major factors shaping the hydrology of the river. The basin area

of 202,000 km^2 represents 25% of the catchment area and 35% of the flow of the Mekong River Basin respectively [20].

Current information and modeling scenarios

Some model-based projections of flood risks in the Lancang-Mekong River continuum predict more frequent and bigger flood events in the future in response to climate change; but through reservoir regulation, flood risks can be mitigated particularly in the upper basin where large reservoirs are located. Models indicate that the combined effects of climate change and reservoir regulation can reduce risks in the upper basin, but the increased flood magnitude and frequency from climate change are still dominant in the lower basin [17]. Recent reports from the Sixth phase of Coupled Inter-comparison Project (CMIP6 models), including the Standardized Precipitation Index (SPI) and Standardized Precipitation-Evapotranspiration Index (SPEI) data, predict that seasonal impacts of droughts in the Lower Mekong River Basin (LMRB) will include wetter rainy season and drier non-rainy season conditions. The SPEI shows that the middle reaches of the LMRB will experience the highest levels of drought duration and intensity [21].

Reservoirs across the Lancang River reduced the annual average flow by 5% at Chiang Saen during 2008-2016, but their impact on flow was undetectable downstream at Vientiane Laos. The streamflow changes noted downstream of Mukdahan in southern Laos, Cambodia, and Vietnam were attributed to local reservoirs and climate change with the latter changing the magnitude and frequency of floods by up to 14% in Cambodia and 45% in South Vietnam [22]. Current data on annual surface water flow in the LMRB indicate a general downward trend

during 1960-2010, with no clear trend after 2010. However, while most studies indicate a decreasing trend, a few studies reported the opposite with increasing flow; this may be the result of different data and methods applied in each study [22].

Hydropower development, irrigation expansion, and global climate change were examined in a study of the LMRB [23]. The authors suggested studies including transboundary collaboration in order to address sustainable development goals that could help to meet planned sustainability objectives. Another study showed that hydropower and irrigation development predicted limited impacts on annual flows under 2040 development scenarios compared to the early development period. ~~However,~~ cli[illegible]e change can reduce the streamflow in June between 2040 to 2080 for some scenarios and increase the streamflow in October in the same period. Furthermore, the streamflow showed significant increases in the long-term period during September-November and December-February but decreases during March-May and June-August. Hydropower decreased the streamflow in the wet season by 7% but increased in the dry season by 29%. The combined impacts of all driving factors caused substantial flow reductions during the first half of the wet season (- 42%) but increased the flow in the dry season by 40% and 14% compared to the early development and nearly current scenario, respectively [24].

A recent study descr[illegible] an integrated approach with mixed methods and assessed future sustainability challenges in social, hydrological, and ecological dimensions using a case study from the Lower Mekong basin. The study area included the combined basin of the Se Kong, Se

San, and Sre Pok (3S) rivers that deliver approximately 20% of the flow to the Mekong River system. The results suggested that future river flows in the 3S river system could move closer to natural conditions during the dry season with increased floods during the wet season and require a shift in the current dam operations, from maintaining minimum flows to reducing flood hazards [25].

The projected changes in river flow trends in the Lancang- Mekong continuum will lead to major impacts on sediment transport and characteristics along different areas of the river channel and in the dam reservoir areas. The changes are both local and site-specific and the PBCM can help to characterize major determinants of the sediment architecture associated with the observed habitat for *N. aperta* (Fig. 4, 5.) [1]. Snai[illegible]bitat sites along the lower Mekong River can be described as patchy habitats or mosaics that display high intra-and inter-site variability in substrate type, nutrient availability, and primary producer organisms that regulate food sources and water quality. [26][27].

Sediment architecture and water quality are major determinants of the aquatic food web components supporting *N. aperta* populations particularly macrophytes, microorganisms, and macroinvertebrates. The *N*[illegible]*perta* ~~habitat~~ on Khong Island is conditioned by sediment architecture, and particle size distribution is a strong determinant of nutrient type, quantity, and availability to support the primary producer base of the food web that is an important source of food and attachment for snails including *N. aperta* (Fig.3-5; Tables 1 and 2). Muntz [28] studied the decomposition of organic matter above and below dams in the USA and found

decomposition rates were significantly greater in the impoundments than in one free-flowing section of the river but did not find a significant relationship between decomposition and oxygen or temperature. The average temperature was slightly warmer in the impoundments, suggesting a stronger effect of temperature than oxygen on decomposition.

Current studies in China indicate similar general results with pre- and post-impoundment studies of hydrological impacts on sediment transport in the Han River Basin in China that produce a significant quantity of sediment loss downstream [29]. Estimates of sediment transport in the Lancang-Mekong River system are highly variable but reflect conditions prevailing prior to the ongoing construction of a cascade of seven dams on the Lancang, which will trap an estimated 83% of the Lancang basin sediment when complete [30]. Thus, almost half of the total natural sediment load of the Mekong will be lost in the reservoirs of the Lancang, and the sediment load of the Lower Mekong River will consist largely of sediment derived from sources within the Lower Mekong River basin itself. These sediment losses could lead to site-specific spates of hungry water [31] causing *[illegible]perta* ~~habitat changes~~ due to scouring and bank and channel erosion [32]. The important role of paleogeography and paleoclimate on speciation and endemism in isolated populations of Schistosoma vectors of the genus *Bulinus* in Africa has been described [33]. Snail vectors can be sensitive to changes in the ecological communities in which they are embedded. Macrophyte communities provide attachment substrate and nutrients for snails in littoral habitats (Fig 5; Tables 1 and 2). Hydro-morphological alterations influenced the functional diversity and composition of macrophyte communities in the Danube River [34]. Macrophytes were reported to modulate the detritus

cycle and change the nutrient supply in *S. mansoni* snail vector habitats [35]. Qualitative and quantitative changes in nutrients can result in significant changes in habitat structure. The enhanced release, export, and transport of diffuse nutrients from watershed forest litter in the Lancang River basin with climate change have been reported using remote sensing data, experimental parameters, and the SWAT model [36].

Tomczyk [37] compared the points closest to and downstream of a Hydro Powerplant (HP) in Poland and noted an increase in concentrations of most heavy metals. Fine-grained fractions dominate the clays and sandy clays upstream of the hydropower plants, while sands, sandy clay loams, or sandy clays are dominated downstream. The dominance of fine-grained fractions upstream of the HPs favors the accumulation of metals due to the high sorption capacity of these fractions. When comparing the analyzed groups of points, the average pH values are arranged in the following sequence: upstream HP (U) > downstream HP (D). EC has opposite values than pH (i.e., U < D) EC is greater downstream. In the 1972 pre-impoundment studies, the same trend in pH and EC was noted between Ban Dan and Khong Island. The Tomcyzk study may support sustainable sediment management options and allow for the development of recommendations related to the rational management of HPs.

Shi (38) developed a niche model using machine learning algorithms to predict the probability of suitable snail habitats for the S. ja[illegible]um vector *Oncomelania hupensis* in China. The six greatest contributors to predicting potential *O. hupensis* distribution included silt content (13.13%), clay content (10.21%), population density (8.16%), annual accumulated temperatures

of ≥0°C (8.12%), night-time lights (7.67%), and average annual precipitation (7.23%). The 1972 baseline studies reported somewhat similar trends in *N. aperta* ~~habitat~~ regarding the percent sand, silt, and clay at Ban Dan and Khong Island (Fig 4).

Reports describing the Lower Mekong Basin indicate that while overall water quality was good up into the 2000s [20][1]. Recent studies indicate that localized degradation has occurred over the past decade near Vientiane City; the Sekong, Sesan, and Srepok Rivers; the Tonle Sap lake system; and the Mekong Delta [39][40]. Water quality degradation likely corresponds to flow alteration, erosion, sediment trapping, and point and nonpoint wastewater.

A study of the relationship between climatic, hydrological, and water quality parameters of the lower Mekong River flowing through four different countries (Thailand, Cambodia, Lao PDR, and Vietnam) indicated that DO, pH, conductivity, Ca, Mg, Na, K, alkalinity, Cl, $SO4^{2-}$ and Si had fair to strong negative correlations with all hydrological parameters. TSS, alkalinity, and conductivity were proposed as sensitive water quality parameters for monitoring the impacts of changing climate in the lower Mekong River [10].

Current reports describing the overall mainstream water quality and ion analyses in the Lower Mekong basin near Khong Island cite good to excellent conditions similar to the monsoon cycle and diel cycle data reported earlier (Figures 3-5) [1][20], however, site-specific data were not collected as part of these surveys and other reports [20][39][40] cite water quality and habitat deterioration at some sites near Khong Island. A river in France reports that dams did not

disturb the seasonality of major anions but did modify silica and phosphorus concentration-flow relationships, especially during low flow. Such changes in the dynamics of river-water composition may affect downstream biological communities [41] and may occur in the LMRB.

Three types of Green House Gases (GHGs), carbon dioxide, methane, and nitrous oxide emitted from reservoirs have an important but often ignored impact on climate change and need to be studied in detail in the LMRB. The biogeochemical cycle of carbon in the aquatic environment is understood to be the main mechanism for generating reservoir-based GHG emissions [42].

The impact of temperature change o[illegible]*aponicum* ~~infections~~ in China was studied using a growing degree-days simulation model. The results predicted changes in transmission patterns and altered frequency and transmission dynamics of *S. japonicum* with climate change. [43].

Kalinda [44] used a synthesis of 19 published articles and indicated that temperature rise may alter the distribution, and optimal conditions for breeding, growth, and survival of intermediate host snails which may eventually increase the spread and/or transmission of schistosomiasis. Other studies in rivers and lakes have reported that transmission may be high at 15-19 ^{o}C. and 20-25°C. respectively [45]. Li [(2] reported that the LMRB region experienced increasing trends for both daytime and nighttime temperatures except for small portions of Thailand and Vietnam. They predict an annual 0.0876°C increase in surface temperature for every 1% decrease in tree density in forest-degraded regions, a substantial annual carbon productivity increase can be expected in reforested regions.

Liu and Xu (46) studied a unique form of flow regulation (e-flow) called hydrospeaking that introduces frequent, short-duration, artificial flow events to river systems in China. They reported that hydropeaking drove plant species distribution, assemblage structure, and species richness-biomass relationships. Hydropeaking also generated complex species-specific effects on plant biomass patterns. They concluded that hydropeaking is likely a major driver of plant community assembly in hydropeaking-affected rivers.

Twenty sites were sampled along the length of the Mekong River from northern Thailand to Lao PDR, Cambodia, and southern Vietnam. Data were collected on four groups of organisms considered suitable for biomonitoring studies: benthic diatoms, zooplankton, littoral macroinvertebrates, and benthic macroinvertebrates. Results indicated that all groups were diverse and abundant in the Mekong River system with considerable variability in abundance and composition from site to site, with the mosaic conditions as described in [7][28] In another study, thirty-two sites were assessed and classified as to their ‘ecological health’ using indicator characteristics including physical and chemical measures, diatoms, and littoral macroinvertebrates and then classified as excellent, good, moderate, or poor. Ten sites were moderate and one site was poor with the remaining sites classified as good to excellent [47].

Limpanont et al. [48] reported a new habitat site for the γ- strain of *N. aperta* (with molecular confirmation of identity) from Nong Khai Province, where it occurred in a habitat novel for this species: under paving slabs instead of under natural bed rocks as noted in (S1) The new site had

agal aufwuchs and was located on the islet in the middle of the Mekong River. The new location is approximately four hundred km upstream from the nearest previously known site for this species.

Discussion

Suggested variables and habitat models to calibrate and update the PBCM are currently available or under development. Water management and economic development plans should fully consider e-flow recommendations that include specific criteria linked to ecological functions and processes [49][50]. A robust approach that includes specific combinations of biological, physical, and chemical data could provide a useful tool to help manage river flows along different impounded areas of the Lancang-Mekong River. Assessments of e-flow should be appropriate for the context and as simple as possible. E-flow regimes should be spatially and temporally specific, connected to ecological processes and functions, and adaptive to changing conditions. One good way of accomplishing this is to develop habitat models that incorporate desired e-flow regime characteristics in the model design. Yarnell and Thoms [51] propose the application of Functional Flows using a flow-chain model and provide two case study examples from Australia and the United States, where improvements in channel habitat and reconnection with the floodplain help to achieve the desired functionality of environmental flows.

A recent case study reported the use of e-flow models coupled with e-flow allocations for a tropical reservoir system by Integration of water quantity (SWAT) and quality (GEFC, QUAL2K) models [52]. The coupled models allowed control of DO and other important water quality

parameters that could influence snail habitat and distribution. Coupled e-flow- ecological niche habitat models could provide a powerful tool for estimating the potential habitat areas that exist in the highly diverse shoreline mosaic of the Lancang-Mekong watershed. It may be possible to create predictive models describing suitable habitats for *N. aperta* and other Schistosoma snail vectors along shoreline areas of the Lancang- Mekong River continuum. The models could be powerful management tools that combine e-flow and habitat models describing water quality with Schistosome biomarkers currently under development [53] in a One Health context to predict possible areas suitable for disease transmission.

Hydro-ecological modeling in SRH-2D and CASiMiR, utilizing flow velocity measurements and macroinvertebrate sampling data should be considered as one method to design flow regimes that protect biological communities and should include hydrological data reflecting human influences and climate and their effects on sediment distribution [54][55].

An example of a coupled e-flow-habitat model could be to combine the ecological niche model applied to characterize the spatial and temporal distribution of *Oncomelania hupensis*, the snail vector of Schistosoma japonica in China as described by Shi et al. [38] with a new model for predicting the terminal settling velocity and drag coefficient of *Oncomelania* for use in hydraulic schistosomiasis control [56].

That model could then be integrated with observations from current e-flow studies and habitat models [57][58][59][60]. Another approach could involve coupling habitat suitability models (HSMs) with frameworks for e-flow estimation models. Szalkiewicz [61] reported a comparative assessment of e-flow values for hydrobiid snails and other macroinvertebrates at two reaches of a river with different levels of hydro morphological change. They noted strong dependence of e-flows on morphological conditions and that the lower limits of e-flows noted were greater than values based on hydrology. Coupling the HSM model with data from the framework approach including information from data-poor river systems [14] could enhance the value of both approaches and provide a useful predictive tool for estimating river ecological habitats in rivers including data-poor systems.

Value and potential applications of the PBCM.

The PBCM framework is presented in (Fig. 6). The model can serve as a template to summarize, compare, and combine pre-impoundment baseline data describing habitat supporting [illegible] *mekongi* ~~transmission~~ by *N. aperta.* The PBCM can help to select/predict which shoreline areas along the Mekong River could support *N. aperta* and other vector snails as well as habitats that will support and protect the biodiversity of the river molluscan community in general. Niche models and habitat suitability models can be powerful tools when combined with current e-flow models (Fig. 6). Together, the models can help to identify effective e-flow strategies to help with the control and management of waterborne disease transmission. Luo [62] completed a recent systematic review and geospatial analysis of schistosomiasis in Southeast Asia and reported that although the prevalence has been substantially reduced since 2000,

focal areas with high prevalence remain. They identified areas including Khong Island, Laos that should be targeted for interventions to reduce disease. The PBCM of *N. aperta* describing habitat during transmission of *S. mekongi* on Khong Island, Laos can be adapted to predict the potential for favorable snail vector habitat along shoreline areas of the Mekong River during climate change. The adapted model could be built using general descriptions of the littoral zone in Davidson [28]. Models of this type have been described and suggested for other data-deficient rivers Sharma [13]; Brierley [12].

It may be possible to create a suite of predictive models describing suitable habitats for *N. aperta* and other Schist[illegible]a snail vectors along the Lancang- Mekong River continuum shoreline areas. The models could be powerful management tools that combine e-flow models describing key water quality parameters with Schistosome biomarkers currently under development in a One Health context to select possible areas of the river likely to support disease transmission.

A Maximum Entropy (Maxent) model predicted suitable habitats for *S. haematobium and S. mansoni* in Africa and was considered to be a robust snail suitability model that may be useful in developing control and management programs for schistosomiasis in the study area [63]. A Bayesian network model (sBN) delineated exposure areas of *S. japonicum* and reported high values of probability of exposure corresponded to polygons where snails could potentially be present. The schistosomiasis exposure sBN could be used by local disease control teams to identify areas of exposure and improve the efficiency of mass drug administration [64]. A study

to address how snail hosts and their interaction with *S. mansoni* influence model predictions indicated that model outputs such as schistosome prevalence in human and snail populations respond to the inclusion of snail age structure [65]. Another study linked quantitatively hydrological drivers to distinct population dynamics through specific density feedbacks and showed that model averaging based on statistical methods yielded reliable projections of snail abundance [66]. The importance of hydrological drivers of habitat structure is evident in snail habitat models in general. A recent study of projected changes in annual extreme rainfall and high and low streamflow events over Southeast Asia under extreme climate change indicates dramatic fluctuations as predicted by a High-resolution Model Intercomparison Project (HighResMIP) multi-model experiment for the period 1971-2050 [67]. Some of the model predictions correspond with areas near the 1972 studies in Laos described in this paper and could influence future [illegible]*perta/S. mekongi* ~~habitat dynamics~~.

References

1. Lanza G. Eutrophication: Causes, Consequences, and Control. In: Ansari AA, Lanza G, Gill SS, Rast W, editors. Eutrophication: causes, consequences an control. Dordrecht: Springer; 2011. p. 373–86.

2. Li Z, Sun Z, Sun L, Liu J, Xiong W, Dong H, et al. Hydrological variation and hydro-sediment interrelation of the Luozha River in the Lancang River Basin. Water Supply. 2022 Apr 18;
3. Kitikoon V, Schneider C, Sornmani S, Harinasuta C, Lanza G. Evidence of the Natural Transmission of Schistosoma japonicum, Mekong strain, at Khong Island Laos. Southeast Asian Journal of Tropical Medicine. 1973;2:350–8.

4. Blair D, Xu Z-B, Agatsuma T. Paragonimiasis and the Genus Paragonimus. Advances in Parasitology. 1999;113–222.
5. Shimada M, Kato-Hayashi N, Chigusa Y, Nakamura S, Ohmae H, Sinuon M, et al. High susceptibility of Neotricula aperta gamma-strain from Krakor and Sdau in Cambodia to Schistosoma mekongi from Khong Island in Laos. Parasitology International. 2007 Jun;56(2):157–60.

6. Institut Pasteur du Laos [Internet]. www.pasteur.la. [cited 2022 Dec 3]. Available from: http://www.pasteur.la
7. Kumagai T, Matsumoto-Takahashi ELA, Ishikawa H, Keomalaphet S, Khattignavong P, Soundala P, et al. Detection of Schistosoma mekongi DNA in Human Stool and Intermediate Host Snail Neotricula aperta via Loop-Mediated Isothermal Amplification Assay in Lao PDR. Pathogens. 2022 Nov 24;11(12):1413.
8. Junk W, Blayey P, Sparks R. The Flood Pulse Concept in River-Floodplain Systems. Canadian Special Publication Fisheries Aquatic Sciences 106; 1989 p. 10pp.
9. Poff NL, Allan JD, Bain MB, Karr JR, Prestegaard KL, Richter BD, et al. The Natural Flow Regime. BioScience. P1997 Dec;47(11):769–84.
10. Prathumratana L, Sthiannopkao S, Kim KW. The relationship of climatic and hydrological parameters to surface water quality in the lower Mekong River. Environment International. 2008 Aug;34(6):860–6.
11. Fan H, He D, Wang H. Environmental consequences of damming the mainstream Lancang-Mekong River: A review. Earth-Science Reviews. 2015 Jul;146:77–91.

12. Poff NL. A river that flows free connects up in 4D. Nature. 2019 May;569(7755):201–2.
13. Brierley GJ, Fryirs KA. River futures : an integrative scientific approach to river repair. Washington, DC, Tucson, Ariz.: Island Press ; Society for Ecological Restoration International;
14. Sharma U. Effective framework for Environmental-flows estimation for data deficient Indian rivers. Journal of Applied and Natural Science. 2019 Jun 10;11(2):545–55.
15. Upatham E. Field Studies on the Bionomics of alpha and gamma races of Tricula aperta in the Mekong River at Khemmarat Ubol Ratchanthani province, Thailand. Malacology Review. 1980;2:239–61.
16. Nishina M. The Composition of the Mekong River Silt and its Possible Role as a Source of Plant Nutrients in the Delta [Internet] [MSc]. [University of Hawaii]; 1974 [cited 2022 Mar]. p. 1–109. Available from: https://scholarspace.manoa.hawaii.edu/handle/10125/56378
17. Wang W, Lu H, Ruby Leung L, Li H-Y, Zhao J, Tian F, et al. Dam Construction in Lancang-Mekong River Basin Could Mitigate Future Flood Risk From Warming-Induced Intensified Rainfall. Geophysical Research Letters. 2017 Oct 25;44(20):10, 378–310, 386.
18. Khir Alla YM, Liu L. Impacts of Dams on the Environment: A Review. International Journal of Environment, Agriculture and Biotechnology. 2021;6(1):064–74.
19. Lauri H, de Moel H, Ward PJ, Räsänen TA, Keskinen M, Kummu M. Future changes in Mekong River hydrology: impact of climate change and reservoir operation on discharge. Hydrology and Earth System Sciences. 2012 Dec 5;16(12):4603–19.
20. Commission (MRC) MR. Mekong River Commission (MRC) [Internet]. www.mrcmekong.org. Available from: https://www.mrcmekong.org
21. Dong Z, Liu H, Baiyinbaoligao, Hu H, Khan MYA, Wen J, et al. Future projection of seasonal drought characteristics using CMIP6 in the Lancang-Mekong River Basin. Journal of Hydrology. 2022 Apr;127815.
22. Yun X, Tang Q, Wang J, Liu X, Zhang Y, Lu H, et al. Impacts of climate change and reservoir operation on streamflow and flood characteristics in the Lancang-Mekong River Basin. Journal of Hydrology. 2020 Nov;590:125472.

23. Liu J, Chen D, Mao G, Irannezhad M, Pokhrel Y. Past and Future Changes in Climate and Water Resources in the Lancang–Mekong River Basin: Current Understanding and Future Research Directions. Engineering. 2021 Sep;
24. SAVATHDY P, KINOUCHI T, KHANOPHET P. ANALYSIS OF WATER INFRASTRUCTURE DEVELOPMENT AND CLIMATE CHANGE IMPACT ON THE STREAMFLOW IN THE LOWER MEKONG RIVER BASIN. Journal of Japan Society of Civil Engineers, Ser B1 (Hydraulic Engineering). 2021;77(2):I265–70.
25. Mohammed I, Bolten J, Souter N, Shaad K, Vollmer D. Assessment of water resources conservation and sustainable management strategies in the Lower Mekong River Basin. 2022 Mar 27;
26. Cullum C, Brierley G, Thomas M. The Spatial Organization of River Systems. In: Brierley G, Fryirs K, editors. River Futures an Integrative Scientific Approach to River Repair. Island Press; 2008.
27. Biomonitoring of the lower Mekong River and selected tributaries [Internet]. Mekong River Commission. Mekong River Commission; 2008 [cited 2021] p. 1–91. Available from: https://mrcmekong.org
28. Muntz O. Decomposition of Organic Matter Above and Below Dams [Internet]. [Eastern Michigan University]; 2022 [cited 2022]. Available from: https://commons.emich.edu/honors/726
29. Jaing C, Wang J, Li C, Wang X, Wang D. Understanding the hydropower exploitation's impacts through a lens of change bin flo-sediment relationship: a case study in the Han River Basin, Chia.
30. Kondolf GM, Rubin ZK, Minear JT. Dams on the Mekong: Cumulative sediment starvation. Water Resources Research. 2014 Jun;50(6):5158–69.
31. Kondolf GM. PROFILE: Hungry Water: Effects of Dams and Gravel Mining on River Channels. Environmental Management [Internet]. 1997 Jul 1;21(4):533–51. Available from: https://people.wou.edu/~taylors/g407/kondolf_97.pdf
32. Rubin ZK, Kondolf GM, Carling PA. Anticipated geomorphic impacts from Mekong basin dam construction. International Journal of River Basin Management. 2014 Dec 11;13(1):105–21.
33. Tumwebaze I, Clewing C, Chibwana FD, Kipyegon JK, Albrecht C. Evolution and Biogeography of Freshwater Snails of the Genus Bulinus (Gastropoda) in Afromontane Extreme Environments. Frontiers in Environmental Science. 2022 May 17;10.
34. Vukov D, Ilić M, Ćuk M, Igić R. The Effect of Hydro-Morphology and Habitat Alterations on the Functional Diversity and Composition of Macrophyte Communities in the Large River. Frontiers in Environmental Science. 2022 Apr 6;10.
35. Desautels DJ, Hartman RB, Shaw K, Maduraiveeran S, Civitello DJ. Divergent effects of invasive macrophytes on population dynamics of a snail intermediate host of Schistosoma Mansoni. Acta Tropica. 2022 Jan;225:106226.
36. Hao X, Ouyang W, Zhang K, Wan X, Cui X, Zhu W. Enhanced release, export, and transport of diffuse nutrients from litter in forested watersheds with climate warming. Science of The Total Environment. 2022 Sep;837:155897.
37. Tomczyk P, Wiatkowski M, Gałka B, Gruss Ł. Assessing the Impact of a Hydropower Plant on Changes in the Properties of the Sediment of the Bystrzyca River in Poland. Frontiers in Environmental Science. 2022 Jul 7;10.

38. Shi Q, Gong Y, Zhao J, Qin Z, Zhang J, Wu J, et al. Spatial and Temporal Distribution Pattern of Oncomelania hupensis Caused by Multiple Environmental Factors Using Ecological Niche Models. Frontiers in Environmental Science. 2022 Jul 12;10.
39. Sor R, Ngor PB, Soum S, Chandra S, Hogan ZS, Null SE. Water Quality Degradation in the Lower Mekong Basin. Water. 2021 May 31;13(11):1555.
40. JUTAMAS SRIPANYA, KWANSIRI RATTANAWILAI, VONGSOMBATH C, VIENGKHONE VANNACHAK, CHUTIMA HANJAVANIT, NARUMON SANGPRADUB. Benthic Macroinvertebrates and Trichoptera Adults for Bioassessment Approach in Streams and Wadeable Rivers in Lao People's Democratic Republic. Tropical Natural History [Internet]. 2022;22:12–24. Available from: https://li01.tci-thaijo.org/index.php/tnh/article/view/254817
41. Fovet O, Ndom M, Crave A, Pannard A. Influence of dams on river water-quality signatures at event and seasonal scales: The Sélune River (France) case study. River Research and Applications. 2020 Mar18;36(7):1267–78.
42. Lu S, Dai W, Tang Y, Guo M. A review of the impact of hydropower reservoirs on global climate change. Science of The Total Environment. 2020 Apr;711:134996.
43. YANG G-J, GEMPERLI A, UTZINGER J, ZHOU X-N, VOUNATSOU P, TANNER M. A GROWING DEGREE-DAYS BASED TIME-SERIES ANALYSIS FOR PREDICTION OF SCHISTOSOMA JAPONICUM TRANSMISSION IN JIANGSU PROVINCE, CHINA. The American Journal of Tropical Medicine and Hygiene [Internet]. 2006 Sep 1 [cited 2020 Nov 16];75(3):549–55. Available from: https://pdfs.semanticscholar.org/081e/2a81bb2d4e49f36c0cb79894390c57712298.pdf
44. Kalinda C, Chimbari M, Mukaratirwa S. Implications of Changing Temperatures on the Growth, Fecundity and Survival of Intermediate Host Snails of Schistosomiasis: A Systematic Review. International Journal of Environmental Research and Public Health [Internet]. 2017 Jan 1;14(1). Available from: https://www.ncbi.nlm.nih.gov/pmc/articles/PMC5295331/
45. McCreesh, Booth M. The Effect of Increasing Water Temperatures on Schistosoma mansoni transmission [Internet]. scholar.google.com. [cited 2022 May 31]. Available from: http://scholar.google.com/scholar_lookup?
46. Liu X, Xu Q. Hydropeaking impacts on riverine plants downstream from the world's largest hydropower dam, the Three Gorges Dam. Science of The Total Environment. 2022 Nov;845:157137.
47. Dao HG, Kunpradid T, Vongsombath C, Do TBL, Prum S. Report on the 2008 Biomonitoring Survey of the Lower Mekong River and Selected Tributaries [Internet]. Mekong River Commission. Vientiane: Mekong River Commission; 2010 Feb [cited 2021 Apr] p. 69 pp. Available from: https://mrcmekong.org
48. Limpanont Y, Maleewong W, Chusongsang P, Intapan PM, Sadaow L, Limsomboon J, et al. A New Population and Habitat for Neotricula aperta in the Mekong River of Northeastern Thailand: A DNA Sequence-Based Phylogenetic Assessment Confirms Identifications and Interpopulation Relationships. The American Journal of Tropical Medicine and Hygiene. 2015 Feb 4;92(2):336–9.
49. Environmental Flows: Harmony between Humans and Nature. International Water Resources Association. International Water Resources Association; 2022 May.

50. Arthington AH, Kennen JG, Stein ED, Webb JA. Recent advances in environmental flows science and water management-Innovation in the Anthropocene. Freshwater Biology. 2018 Apr 16;63(8):1022–34.
51. Yarnell SM, Thoms M. Enhancing the functionality of environmental flows through an understanding of biophysical processes in the riverine landscape. Frontiers in Environmental Science. 2022 Oct 21;10.
52. Kumar MS, Priya PNC, Shaik R, Singh SK. Environmental Flows Allocation for a Tropical Reservoir System by Integration of Water Quantity (SWAT) and Quality (GEFC, QUAL2K) Models. Water Resources Management. 2022 Oct 15;
53. Uthailak N, Adisakwattana P, Thiangtrongjit T, Limpanont Y, Chusongsang P, Chusongsang Y, et al. Discovery of Schistosoma mekongi circulating proteins and antigens in infected mouse sera. Gasset M, editor. PLOS ONE. 2022 Oct 13;17(10):e0275992.
54. Hough IM, Warren PH, Shucksmith JD. Designing an environmental flow framework for impounded river systems through modelling of invertebrate habitat quality. Ecological Indicators. 2019 Nov;106:105445.
55. Jay DA, Naik PK. Distinguishing human and climate influences on hydrological disturbance processes in the Columbia River, USA. Hydrological Sciences Journal. 2011 Oct;56(7):1186–209.
56. Liu X, Wang J, Chai Z, Min F, Jiang X, Zhu K, et al. New model for predicting terminal settling velocity and drag coefficient of the Oncomelania. Frontiers in Environmental Science. 2022 Nov 2;10.
57. Chen A, Wu M, Chen K, Sun Z, Shen C, Wang P. Main issues in research and practice of environmental protection for water conservancy and hydropower projects in China. Water Science and Engineering. 2016 Oct;9(4):312–23.
58. Chen A, Wu M, Wu S, Sui X, Wen J, Wang P, et al. Bridging gaps between environmental flows theory and practices in China. Water Science and Engineering. 2019 Dec;12(4):284–92.
59. Chen A, Wu M. Managing for Sustainability: The Development of Environmental Flows Implementation in China. Water. 2019 Feb 28;11(3):433.
60. Theodoropoulos C, Georgalas S, Mamassis N, Stamou A, Rutschmann P, Skoulikidis N. Comparing environmental flow scenarios from hydrological methods, legislation guidelines, and hydrodynamic habitat models downstream of the Marathon Dam (Attica, Greece). Ecohydrology. 2018 Aug 23;11(8):e2019.
61. Szałkiewicz E, Kałuża T, Grygoruk M. Environmental Flows Assessment for Macroinvertebrates at the River Reach Scale in Different Degrees of Hydromorphological Alteration. Frontiers in Environmental Science. 2022 Apr 11;10.
62. Luo, C., Wang, Y., Su, Q., Tang, S., Bergquist, R., Zhang, Z., Hu, Y. Mapping schistosomiasis risk in Southeast Asia: a systematic review and geospatial analysis. International Journal of Epidemiology , 2022; 8: 1-13. https://doi.org/1093/ije/dya c 227

63. Manyangadze T, Chimbari MJ, Gebreslasie M, Ceccato P, Mukaratirwa S. Modelling the spatial and seasonal distribution of suitable habitats of schistosomiasis intermediate host snails using Maxent in NDUMO area, KwaZulu-Natal Province, South Africa - Parasites & Vectors [Internet]. BioMed Central. BioMed Central; 2016 [cited 2023Jan21]. Available

Commented [kl1]:

from: htt Anderson LC, Loker ES, Wearing HJ. Modeling schistosomiasis transmission: The importance of snail population structure. Parasites & Vectors. 2021;14(1). ps://parasitesandvectors.biomedcentral.com/articles/10.1186/s13071-016-1834-5

64. Araujo Navas AL, Soares Magalhães RJ, Osei F, Fornillos RJ, Leonardo LR, Stein A. Modelling local areas of exposure to schistosoma japonicum in a limited survey data environment. Parasites & Vectors. 2018;11(1).
65. Anderson LC, Loker ES, Wearing HJ. Modeling schistosomiasis transmission: The importance of snail population structure. Parasites & Vectors. 2021;14(1).
66. Perez-Saez J, Mande T, Ceperley N, Bertuzzo E, Mari L, Gatto M, et al. Hydrology and density feedbacks control the ecology of intermediate hosts of schistosomiasis across habitats in seasonal climates. Proceedings of the National Academy of Sciences. 2016;113(23):6427–32.
67. Hariadi MH, van der Schrier G, Steeneveld G-J, Sutanto S, Sutanudjaja E, Ratri DN, et al. A high-resolution perspective of extreme rainfall and river flow under extreme climate change in Southeast Asia. 2023

Figure 1

Click here to access/download;Figure;F1 Water Qual.tif

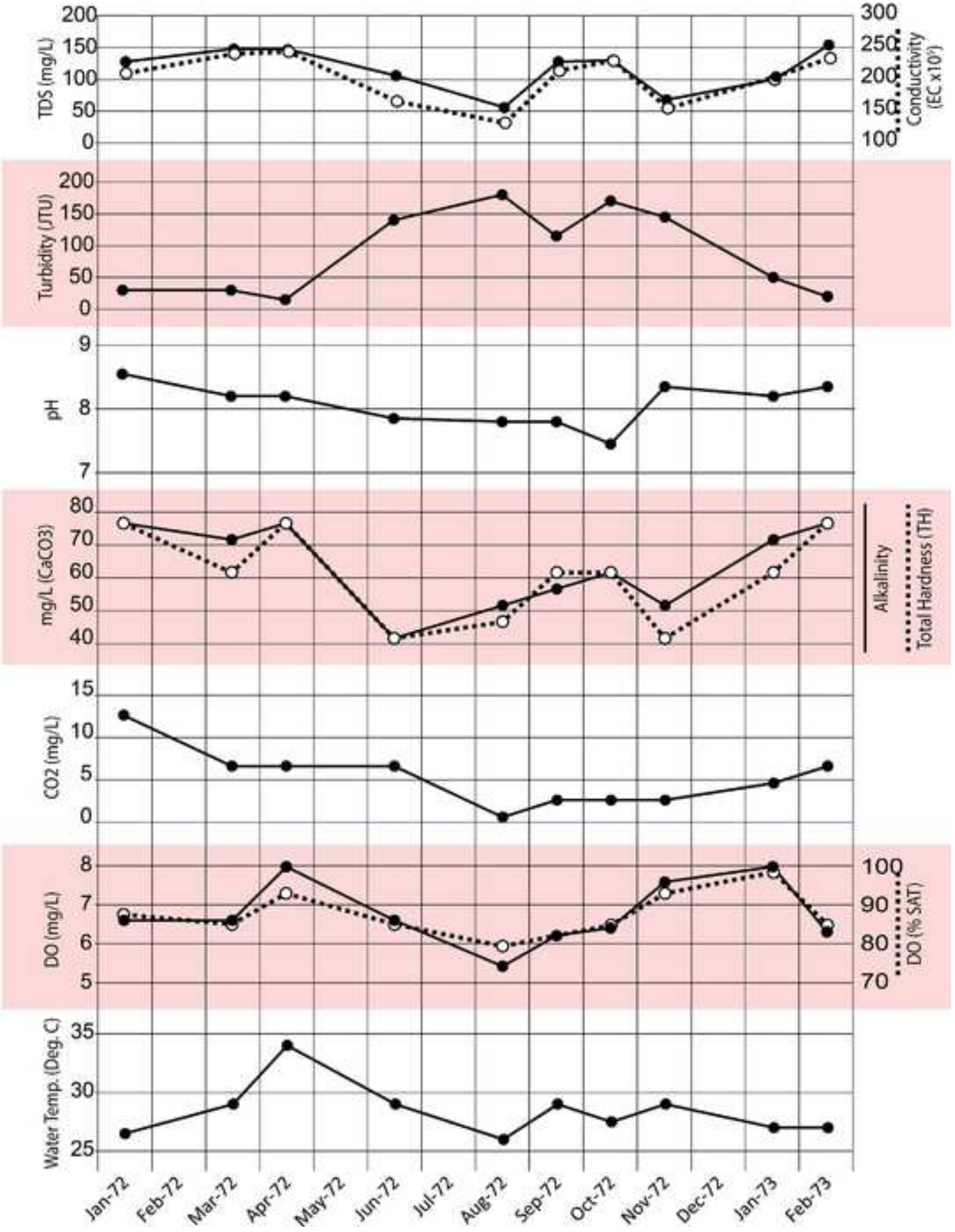

Figure 2

Click here to access/download;Figure;F2 Ions.tif

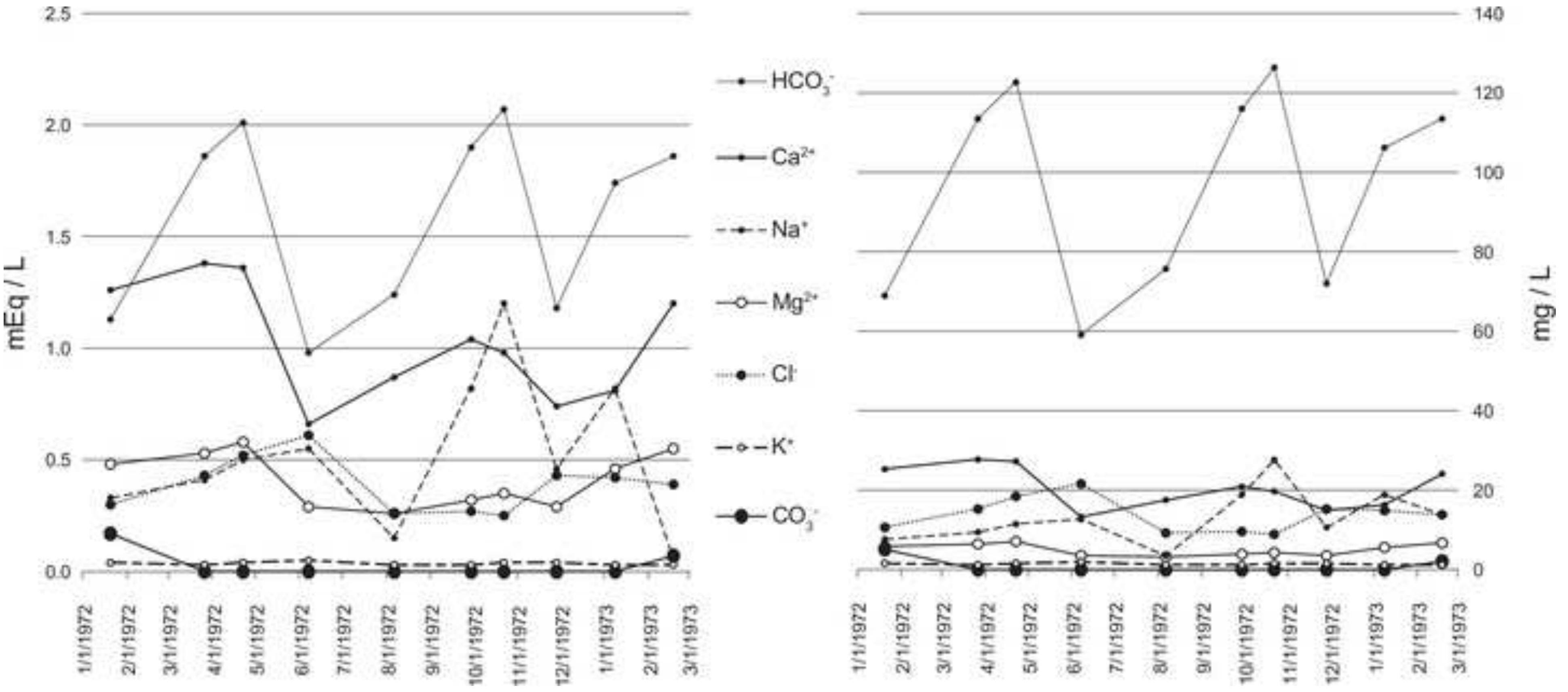

Click here to access/download;Figure;F3 Diel Cycle.tif

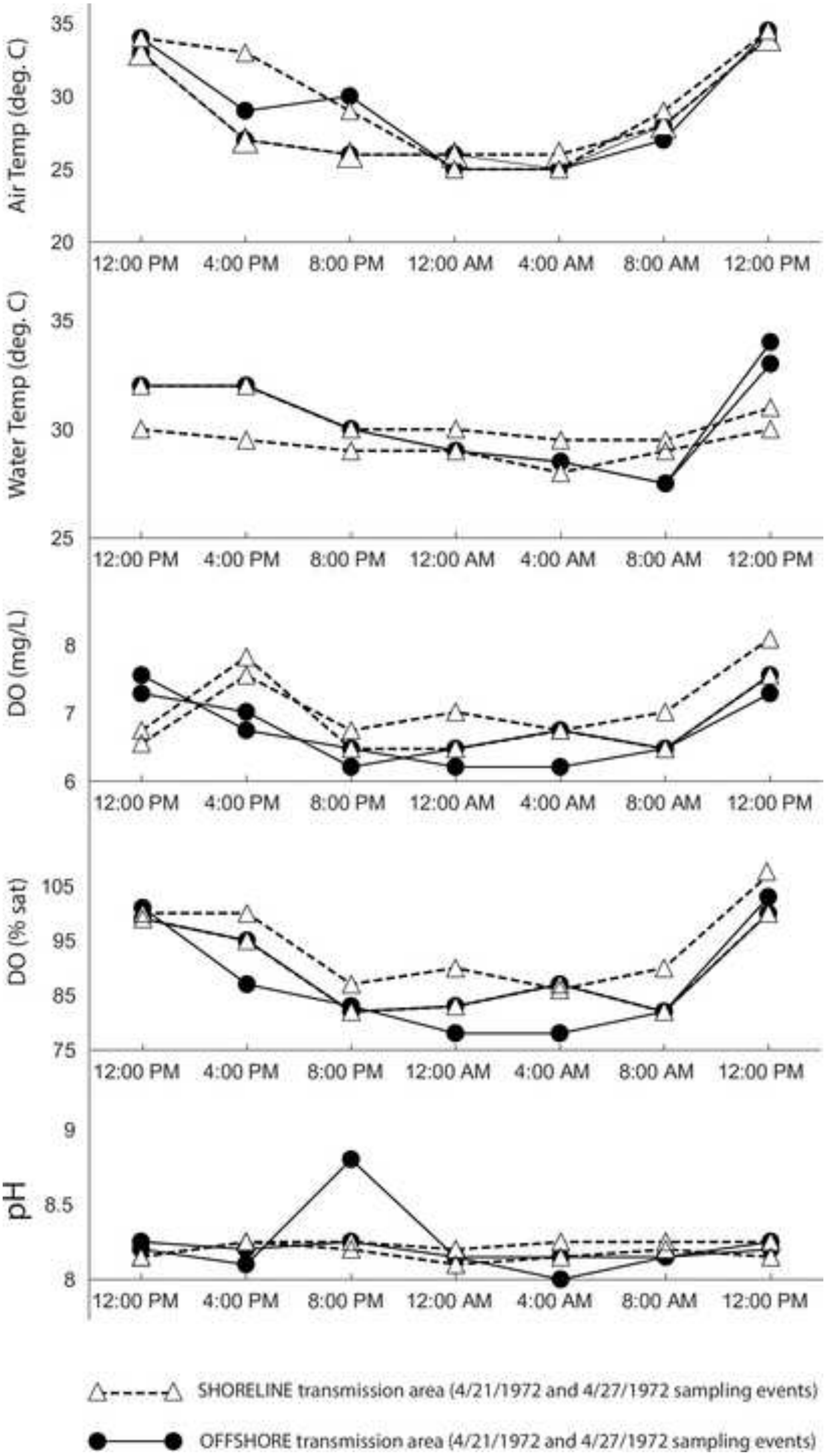

Figure 4

Click here to access/download;Figure;F4 Sediment.tif

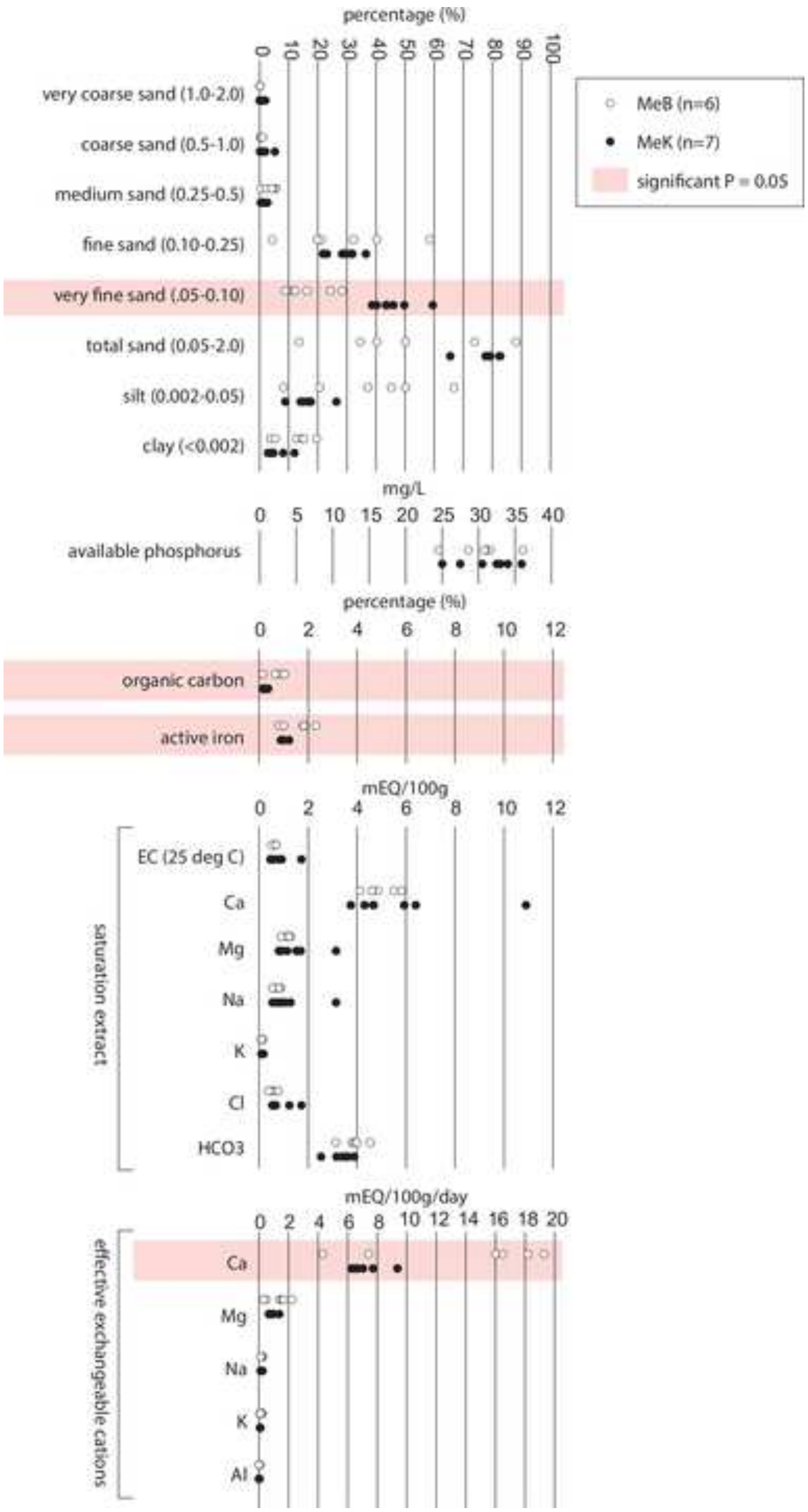

Figure 5

Click here to access/download;Figure;F5 Food Web KI.tif

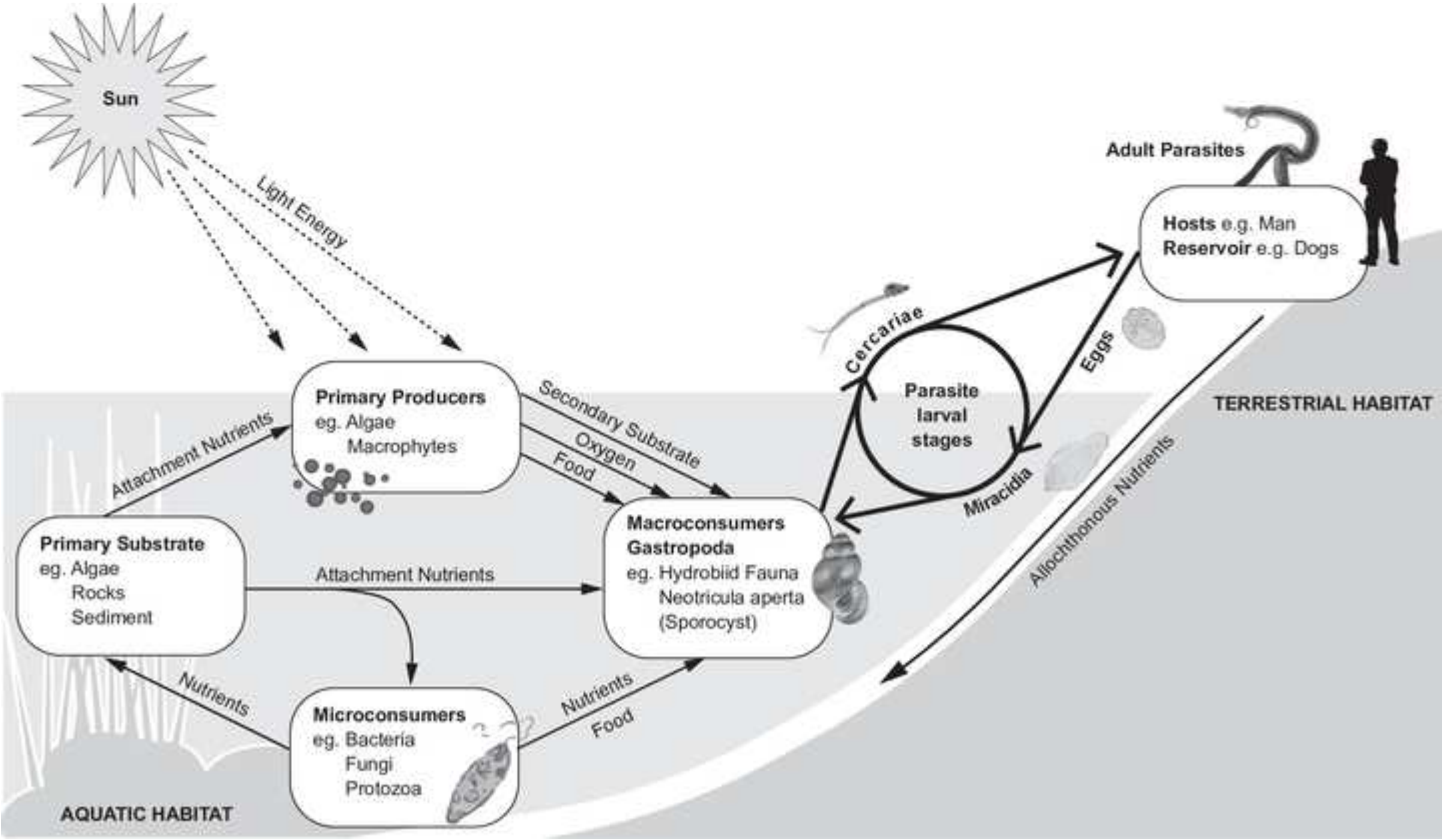

 Click here to access/download;Figure;Fig 6 PBCM.tif

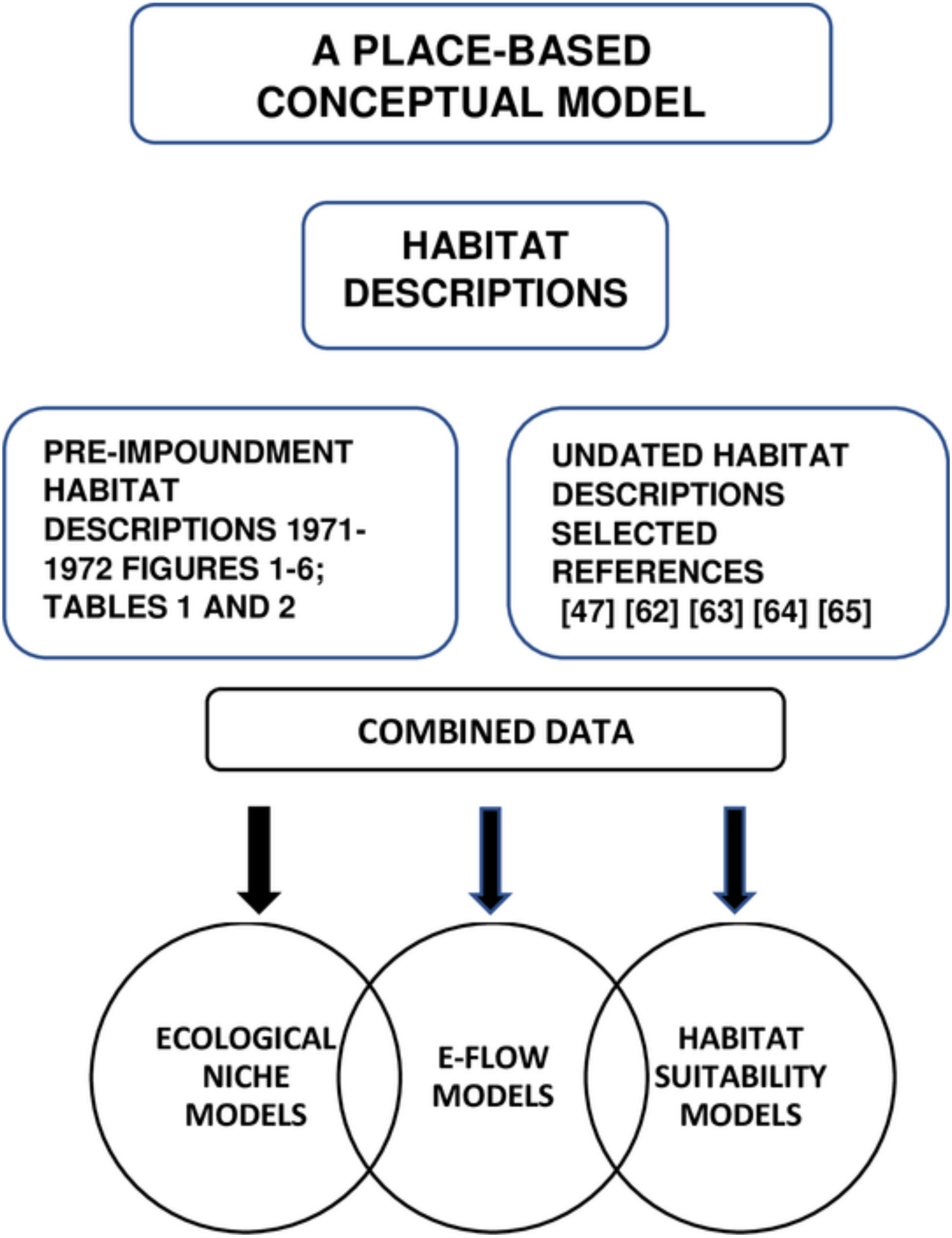

Click here to access/download;Table;Table 1 Algal-Gastropod Association.docx

Table 1

Algal – Gastropod Associations – Mekong River at Khong Island, Laos 1971-73*

Microhabitat	Algal Assemblage	Gastropods on Algae
Algae attached to wood and rock outcroppings or as free-floating mats 5 meters off shore.	*Ulothrix* sp. with dense epiphytic communities of *Malleodendron* sp. and *Navicula* sp.	*Tricula aperta*<br>*Pachydrobia mcmulleni*
Free-floating macrophytes near shorelines.	*Azolla sp.* with epiphytic *Navicula* sp.	*Stenothyra hybocystoides*<br>*S. basisculpta*
Algae attached to submerged twigs: rocks and leaves near shoreline.	*Spirogyra* sp.<br>*Oedogonium* sp.<br>*Ulothrix* sp. with epiphytic *Malleodendron* sp. Twigs with epiphytic *Spirulina* sp. and *Fragilaria* sp. leaves with epiphytic Closterium sp. and *Navicula* sp.	*T. aperta*<br>*S. hybocystoids*<br>*S. basisculpta*<br>*S. cambodiensis*<br>*P. crooki*<br>*P. wykoffi*<br>*P. mcmulleni*

*Collections from Ban Xieng Wang, site of documented Schistosomiasis transmission. See F.4. Kitikoon et al. 1973

Table

Click here to access/download;Table;Table 2 Distribution Patterns.docx

Table 2

Distribution Patterns of Littoral Zone

Gastropods Species, Khong Island, Laos 1972*

Species	Locale**						
	(1)	(2a)	(2b)	(3a)	(3b)	(4a)	(4b)
*Tricula aperta**	+	+	+	+	+	+	+
Stenothyra basisculpta Brant	+	+	+	+	-	+	-
S. hybocystoides Bavay	+	+	+	+	+	+	+
S. laotiensis Temcharoen	-	-	-	-	+	-	-
S. cambodiensis Brandt	-	+	-	-	-	-	-
Stenothyra sp. Juveniles	+	+	-	-	+	+	+
Manningiella expansa Brandt	+	+	-	-	+	+	+
M. pellucida Bavay	+	+	-	-	+	+	+
Pachydrobia mcmulleni[1]	-	+	-	-	-	-	-
P. crooki	-	+	-	-	-	-	-
P. wykoffi	-	+	-	-	-	-	-
Pachydrobiella brevis Bavay	+	+	-	-	+	+	+
Jullienia acuta Poirier	+	-	-	-	-	-	-
J. harmandi Poirier	+	-	-	-	-	-	-
Paraprosothenia iijimai Bandt	+	-	-	-	-	-	-
P. levayi Brandt	-	+	-	-	-	-	-
Lacunopsis fisherpiettei Brandt	-	+	-	-	-	-	+
L. ventricose Poirier	+	-	-	-	-	-	+
Lacunopsis sp. Juveniles	-	+	-	-	-	-	+
Clea (Anentome) helena Philippi	-	+	+	-	-	+	-
Wattebledia crosseana Wittebled	+	-	-	-	-	-	-
Wattebledia sp. Juveniles	-	-	-	-	-	+	-
Hydrorissoia hospitalis Brandt	+	-	-	-	-	-	-
Bithynia sp. Juveniles	-	-	-	-	-	-	+

**Lithoglyphopsis aperta* Temcharoen, vector of human Schistosomiasis

**Numbers in brackets identify east shore locale; a and b are stations collected within a locale. + and – represent present or absent respectively. See F-4 Kitikoon et al. 1973

(1) Military camp area

(2) Ban Xieng Wang, site of documented Schistosomiasis transmission

(3) Dooley Hospital area

(4) Ban Na area

Click here to access/download
Supporting Information
Supplementary Materials.pdf
