# Supplementary material for: A Place-Based Conceptual Model (PBCM) of Neotricula aperta/Schistosoma mekongi habitat before and after dam construction in the Lower Mekong River
Source: PLoS Negl Trop Dis. 2023 Oct 6;17(10):e0011122. doi: 10.1371/journal.pntd.0011122 (PMC10584140; doi:10.1371/journal.pntd.0011122)
Supplement: Attachment — Submitted filename: PNTD-D-23-00131_R1_reviewer.pdf [file pntd.0011122.s004.pdf]

PLOS Neglected Tropical Diseases

A Place-Based Conceptual Model (PBCM) of Neotricula aperta /Schistosoma mekongi habitat before and after dam construction in the Lower Mekong River.

--Manuscript Draft--

**Manuscript Number:**	PNTD-D-23-00131R1
**Full Title:**	A Place-Based Conceptual Model (PBCM) of Neotricula aperta /Schistosoma mekongi habitat before and after dam construction in the Lower Mekong River.
**Short Title:**	A PBCM of Neotricula aperta habitat
**Article Type:**	Research Article
**Keywords:**	Keywords<br><br>Neotricula aperta<br>Schistosoma mekongi<br>Vector snail habitat model<br>Place-Based Conceptual Model (PBCM)<br>E-flow habitat management<br>Lancang- Mekong River<br>Climate change dams neglected tropical disease
**Abstract:**	Abstract<br>In 1971, scientists from Mahidol University in Thailand and the Smithsonian Institution in the USA formed a research team to study a new species of Schistosoma in the Mekong River in Thailand and Laos. The studies were completed during 1971-1973 prior to the construction of any dams or restrictions to the natural flow regime of the Mekong River, and provide a unique description of the natural ecological state of the river that can serve as a baseline for current research. The natural transmission of Schistosoma japonicum, Mekong Strain was first reported on Khong Island, Laos in 1973 using sentinel mice. The first detailed description of the habitat ecology of the snail vector Neotricula aperta was done on-site in 1971 simultaneous with that research and is unique in providing the only description of the river shoreline habitat before any dams were built and any alteration of the natural flow regime was in place. Aggregating current information in a Place-Based Conceptual Model (PBCM) as an organizing template along with current habitat models that combine ecological data with e-flows can be developed and used as a tool to predict suitable habitat for snails. The natural flow regime of the Mekong River prior to any impoundments is described with current updates on the potential impacts of climate change and dams with flow related snail habitat characteristics including sediment drift and water quality. The application of the PBCM to describe and compare descriptive information on current and potential future N. aperta/S. mekongi habitat is discussed.
**Additional Information:**	
**Question**	**Response**
**Government Employee**<br><br>Are you or any of the contributing authors an employee of the United States government?<br><br>Manuscripts authored by one or more US Government employees are not copyrighted, but are licensed under a CC0 Public Domain Dedication, which allows unlimited distribution and reuse of the	No - No authors are employees of the U.S. government.

Powered by Editorial Manager® and ProduXion Manager® from Aries Systems Corporation

article for any lawful purpose. This is a legal requirement for US Government employees.<br><br>This will be typeset if the manuscript is accepted for publication.	
**Financial Disclosure**<br><br>Enter a financial disclosure statement that describes the sources of funding for the work included in this submission and the role the funder(s) played. This includes grants and any commercial funding of the work or authors.<br><br>This statement will be typeset if the manuscript is accepted for publication.<br><br>Review the submission guidelines and the instructions link below for detailed requirements and guidance.	The authors received no specific funding for this work.
**Competing Interests**<br><br>On behalf of all authors, disclose any competing interests that could be perceived to bias this work.<br><br>This statement will be typeset if the manuscript is accepted for publication.<br><br>Review the instructions link below and PLOS NTDs' competing interests policy to determine what information must be disclosed at submission.	The authors have declared that no competing interests exist.
**Data Availability**<br><br>Provide a **Data Availability Statement** in the box below. This statement should detail where the data used in this submission can be accessed. This statement will be typeset if the manuscript is accepted for publication.	All relevant data are within the manuscript.

Before publication, authors are required to make all data underlying their findings fully available, without restriction. Review our PLOS Data Policy page for detailed information on this policy. Instructions for writing your Data Availability statement can be accessed via the Instructions link below.	

Powered by Editorial Manager® and ProduXion Manager® from Aries Systems Corporation

Manuscript Click here to access/download;Manuscript;PLOS JULY 22 docx.docx

A place-based conceptual model (PBCM) of *Neotricula aperta/Schistosoma mekongi* habitat before and after dam construction in the Lower Mekong River

Guy R Lanza[1]*

E. Suchart Upatham[2]

Ang Chen[3]

[1]Corresponding Author - Division of Environmental Sciences, College of Environmental Sciences and Forestry (ESF), State University of New York, Syracuse, NY USA

https://orcid.org/0000-0002-6606-5128

glanza40@gmail.com

[2]Department of Biology, Faculty of Science, Mahidol University, Rama 6 Road, Bangkok, Thailand

edupatham@gmail.com

[3]China Three Gorges Corporation, Wuhan, 430010 China

chenang@foxmail.com

*Corresponding author

E-mail: glanza40@gmail.com (GL)

Abstract

In 1971, scientists from Mahidol University in Thailand and the Smithsonian Institution in the USA formed a research team to study a new species of Schistosoma in the Mekong River in Thailand and Laos. The studies, completed during 1971-1973, prior to the construction of any dams or restrictions to the natural flow regime of the Mekong River, provide a unique description of the natural ecological state of the river that can serve as a baseline for current research. The natural transmission of *Schistosoma japonicum*, Mekong Strain, was first reported on Khong Island, Laos in 1973 using sentinel mice. The first detailed description of the habitat ecology of the snail vector *Neotricula aperta* was done on-site in 1971 simultaneously with that research and is unique in providing the only description of the river shoreline habitat before any dams were built and any alteration of the natural flow regime was in place. Aggregating current information in a Place-Based Conceptual Model (PBCM) as an organizing template, along with current habitat models that combine ecological data with e-flows, can be developed and used as a tool to predict suitable habitats for snails. The natural flow regime of the Mekong River prior to any impoundments is described with current updates on the potential impacts of climate change and dams with flow-related snail habitat characteristics, including sediment drift and water quality. The application of the PBCM to describe and compare descriptive information on current and potential future *N. aperta/S. mekongi* habitat is discussed.

Author Summary

We provide a unique profile of *Neotricula aperta* habitat in the Mekong River in Laos and Thailand during the transmission of *Schistosoma mekongi* prior to the construction of any dams on the river. A Place-Based Conceptual Model (PBCM) is described to help identify potential new areas, with suitable habitats for snails and disease transmission. The natural flow regime of the Mekong River prior to any impoundments is described with current updates on the potential impacts of climate change and flow-related snail habitat characteristics, including sediment drift and water quality. Environmental management strategies are outlined, emphasizing environmental flow (e-flow) and habitat models, with the potential to mitigate *N. aperta* distribution and S. mekongi disease transmission. The PBCM can serve as a template to aggregate and compare historical baseline information (Before dams) with current data (After dams) to create predictive models describing suitable habitats for *N. aperta* and other snail intermediate hosts and vectors along shoreline areas of the Mekong River continuum. The PBCM model could be a powerful management tool when combined with habitat suitability and e-flow models. Adding Schistosome biomarkers currently under development in a One Health context to select possible areas of a river likely to support disease transmission could extend the value of the model.

Introduction

We describe a Place-Based Conceptual Model (PBCM) using pre-and post-dam data from the lower Mekong River in Thailand and Laos. The PBCM describes snail habitats and can be an effective tool to identify potential new foci for *Neotricula operta* and *Schistosoma mekongi*

along the Mekong River as shown in (Figure 1). The Mekong River continuum is approximately 4,909 km long with an average annual discharge of 14,500 m^3/s [1]. The lower Mekong River basin is a key biodiversity hotspot with an invertebrate species array dominated by gastropods. Up-to-date information on shoreline habitat and snail communities is needed to help predict potential disease transmission sites and to conserve invertebrate biodiversity [2][3]. In 1971 scientists from Mahidol University in Thailand and the Smithsonian Institution in the USA formed a research team to study a suspected new species of *Schistosoma japonicum* in the Mekong River in Thailand and Laos. In 1973 the team published the first report of the natural transmission of *Schistosoma japonicum*, Mekong Strain, on Khong Island, Laos (Latitude $14^0 7^/ 30^{//}$N, Longitude $105^0 51^/ 45^{//}$E) using sentinel mice [4]. These studies also described the habitat ecology of *N. aperta* during the transmission of *S. mekongi* in Thailand and Laos before any dams were built on the river, including data on the snail community, plants, algae, bacteria, and water and sediment quality [4][5]. The research provides a unique baseline pre-impoundment (Before Dams) description of the biota, physical parameters, and river water quality. This report provides a Place-Based Conceptual Model (PBCM) that compares information describing the habitat of *N. aperta* during the transmission of S*. mekongi* before dams were built on the Mekong River with the results from more recent post-dam studies of the Lower Mekong River in Laos, Thailand, and Cambodia [5][6]. The model uses current reports describing climate change impacts and altered river flow/sediment transport caused by dams to predict potential future *N. aperta* habitat and suggests management strategies to reduce disease transmission.

Figure 1. Placed-Based Conceptual Model

Climate change can alter the habitat for snails described in the PBCM by driving changes in weather patterns, flow regimes, water quality, sediment transfer, and other key environmental factors. Wang [7] notes that the Lower Mekong River Basin (LMRB) faces two major challenges to its water resources in the 21st century: potential climate change and ongoing and planned dam constructions. Alla [8] reviewed the impacts of dams and noted that they result in major negative impacts on water quality, aquatic ecology, land, terrestrial wildlife, vegetation, and air quality and recommended mitigation measures that should be put in place to reduce these negative impacts on the environment. A changing climate could modify rainfall patterns leading to changes in the hydrological cycle, including shifts in monsoon patterns that influence the water quality and sediments supporting the biota described in the PBCM. One report indicates that within the coming 20–30 years the operation of planned hydropower reservoirs is likely to have a larger impact on the Mekong hydrograph than the impacts of climate change, particularly during the dry season. The report also predicts that climate change will increase the uncertainty of the estimated reservoir operation impacts and that the direction of the flow-related changes induced by climate change is partly unclear [9]. When all the planned dam constructions are complete, the total active capacity of the LMRB is estimated to increase from 5 km^3 to more than 100 km^3. Laos lies almost entirely within the LMRB and its climate, landscape, and land use are the major factors shaping the hydrology of the river. The basin area of 202,000 km^2 represents 25% of the catchment area and 35% of the flow of the Mekong River Basin respectively [10]. The PBCM framework integrates ecological information describing the significance of the flood pulse and the natural flow regime [11,12], with the effects from dams and other barriers that change the natural flow, water quality [13], and sediment distribution.

Most of the fragmentation and disconnection caused by the world's 2.8 million dams is due to structures associated with reservoir areas greater than 1000 m^2 [14]. The PBCM framework has value in locating potential shoreline sites along the Mekong River that could provide habitat for snail intermediate hosts and vectors including *N. aperta.*

Methods

Fig 2. Is a map showing the study sites in Ban Dan, Thailand, and Khong Island, Laos. On-site water chemistry included water quality and ion measurements over one monsoon cycle in Thailand and Laos (Figs 3- 5). Diel cycles were completed on two dates during schistosome transmission with measurements of air temperature, water temperature, dissolved oxygen (DO), percent saturation and hydrogen ion concentration (pH) (Figs 6 and 7). Two stations were examined: one near shore with reduced current, extensive substrate, and snail communities and the other approximately 10 m offshore with swift current, very limited substrate, and no visible snail communities. Turbidity, total dissolved solids, electroconductivity, carbon dioxide, alkalinity, total hardness, calcium, magnesium, potassium, sodium, bicarbonate, and carbonate levels were measured at 2 diel intervals, 12 noon and midnight. A Model DR-EL portable water engineering kit (Hach Chemical Company) was used for on-site measurements. Additional chemistry was done in Bangkok on water and sediments by the water analysis laboratory of the Thai Department of Land Development, Ministry of Agriculture and Cooperatives following APHA procedures. Sediment analyses included particle size, organic carbon, available phosphorus, active iron, conductivity, soluble ions, and Cation Exchangeable Capacity (CEC). A

2-sample t-test for which the variances were not assumed to be equal was used for statistical analyses to compare mean values of 23 sediment parameters at Ban Dan, Thailand, and Khong Island, Laos (Fig 8). Biological samples were collected from natural and artificial substrates. Identification of aquatic plants, algae, and bacteria was done by the Biology Department at Khon Kaen University, and snail identification was done by the Faculty of Tropical Medicine and Hygiene at Mahidol University. Details of the methods used are provided in [3,4] (Figs 9 and 10).

The PBCM was built following methods developed by Brierley and Fryirs [15], and Sharma [16] using Before and After Dam habitat descriptions. *N. aperta* has also been reported as a host for *Paragonimus veocularis* and experimentally as a host for *P. heterotremus* [17].

The PBCM is presented in Fig. 1, with historical 1971-1973 information (Before Dams) and updated information from 9 Lower Mekong River (LMR) sites in Laos, Thailand, and Cambodia surveyed by the Mekong River Commission during 2008-2010 (After Dams) [5,6]. The model includes site-specific changes after dams were built including habitat similarity and differences with pre-dam conditions regarding sediments, water quality, and biota.

Fig 2. Map of the study area

Fig 3. Habitat description and food web

Fig 4. Water quality one monsoon cycle

Fig 5. Water ions one monsoon cycle

Fig 6. 24-hour (diel) cycle April 21, 1972

Fig 7. 24-hour (diel) cycle April 27, 1972

Fig 8. Sediment characteristics

Fig 9. Algal-snail associations

Fig 10. Snail species

Results

PBCM Application

Historical descriptions of *N. aperta* habitat (Before Dams 1971-73)

The historical information from the 1971-73 research (Before Dams) is provided in Fig 1-10.

The importance of water flow on the infection rate of *S. mekongi* was highly evident during pre-impoundment baseline studies. Although specific measurement of flow was not part of these studies, shoreline areas with relatively lower flow and frequent contact by humans and animals at Khong Island, Laos were associated with higher infection rates of *S. mekongi.* Offshore areas in deeper water with moving currents are relatively free of human contact and the mixing, dilution, and lack of substrate for attachment for *N. aperta* may explain the absence of significant infection (Figs. 9-10). Upatham [18] studied the Mekong River in Thailand and Laos and described the association of different water flow patterns on the bionomics of hydrobiid snails, including the alpha- and gamma-strains *N. aperta*. He reported higher densities of snails in areas with higher water flow, but data were not collected on the transmission of *S. mekongi* [18]. Site studies of favorable habitat for N. aperta at Khong Island prior to any changes in the natural flow regime of the Lower Mekong River indicate that the site reflected similar

downstream water quality and sedimentation characteristics when compared to an upstream reference site at the village of Ban Dan, Thailand (Fig 2, Fig 4, Fig 5, Fig 8).

Water quality and water ion data from one monsoon cycle in the Mekong River prior to impoundment indicated a cyclic but stable habitat for snails, algae, and aquatic plants (Fig 4 and 5). Sediment data indicated lower levels of some parameters due to particles settling out with distance (Fig. 3). Data from other studies of downstream stations in Vietnam showed similar trends in sediment transport and characteristics [19]. Statistically significant differences were noted in very fine sand, organic carbon, active iron, and Ca^{2+} (Fig 8). Studies of substrates favored by *N. aperta i*ndicate that they use hard substrates such as tree branches, leaves, metal, rocks, and aquatic plants (3) and that sand was seldom used by *N. aperta* (Fig 3, 9, 10,). Sediments are also a major source of nutrients supporting the primary producer community that provides substrate and food for *N. aperta* and other snail species of the Lower Mekong River. The microbial primary producer community on Khong Island had sixteen species that serve as food and substrate for *N. aperta* (Figs 9 and 10) and twenty-three species of dominant snails (Fig 10). The potential role of snail predation by fish, turtles, and other aquatic organisms is a topic that needs study in the *N. aperta* habitat areas. A study of the use of fish to control parasitic diseases, including schistosomiasis in Lake Malawi, identified the challenges involved in biological control. [20] Mekong River fish that are snail predators include (*Yasuhikotakia morleti)* [21] and *Pangasius conchophilis.* [22] The snail-eating turtle *Malayemys subtrijuga* in the Mekong River in Vietnam is known to be infected by 2 digenean species and could impact snail populations in different river areas. [23] The anticipated changes described in the updated

N. aperta habitat descriptions could affect fish and turtle populations in the study area with impacts on snail predation.

Current description of potential *N. aperta* habitat in the LMR (After Dam surveys) of 2008-2010.

The MRC survey data used in the PBCM identified several basic similarities and differences in the habitat comparisons between 1971-73 and 2008-2010 [5,6]. Our examples of applying the PBCM to predict potentially suitable habitats for *N. aperta* and other snails compares data from recent surveys along with the historical baseline data from 1971-73. Recent reports of mainstream water quality were described as very good overall, but some areas with degraded habitat and water quality were reported. [5][6]. Although *N. aperta* was not reported from the surveys, one site surveyed at Done Ngiev, Laos was very similar to the Khong Island Laos transmission site with sand substrate, *Homonia riparia* shrubs, and filamentous algae and diatoms. The PBCM identified the Done Ngiev site as a good match to Khong Island and would be a good site for more detailed follow-up habitat studies. Another similar site in Cambodia, at Kampi pool near the Lao border, had a sand substrate with some stones and a snail population of almost 400 *Stenothra sp*., a common co-habitant of *N. aperta*. The PBCM also identified sites in Laos, Thailand, and Cambodia that are dissimilar to Khong Island and not good matches. Sites at Ban Xiengkok, Laos, Nakorn Panom, Thailand, and Se San, Cambodia had substrates dominated by rocks, gravel, and mud without much sand and generally low water quality and turbulent currents.

Shimada [24] described the high susceptibility of the gamma strain of *N. aperta to S. mekongi* from Khong Island, Laos. A 2017 survey from Khong Island noted the presence of more than 5500 *N. aperta* snails from the same shoreline areas studied in 1972, with some snails testing positive *for S. mekongi* [25]. More recently, Kumagai [26] developed a loop-mediated isothermal amplification (LAMP) assay for *S. mekongi* and evaluated the utility of the assay for detecting DNA of *S. mekongi* in human stool and snail samples in endemic areas on Khon Island, Laos. The LAMP assay results were used to develop a risk map for monitoring *S. mekongi* and preventing epidemics. Although these surveys [25,26] did not include the site habitat characteristics, the results strongly suggest that some of the favorable *N. aperta/S mekongi* habitats outlined in the Before Dam descriptions remain in place at Khong Island. A follow-up detailed study of the Khong Island site could provide new information to update the PBCM. The PBCM will benefit from additional updates that provide information on current environmental trends, particularly the impacts of climate change and dam construction and operation.

Models characterizing general impacts from climate change and dams in the study area.

Models describing climate change impacts on the LMR can be useful in updating information in the PBCM. Some model-based projections of flood risks in the Lancang-Mekong River continuum predict more frequent and bigger flood events in the future in response to climate change. Through reservoir regulation, flood risks can be mitigated particularly in the upper basin where large reservoirs are located. Models indicate that the combined effects of climate change and reservoir regulation can reduce risks in the upper basin, but the increased flood

magnitude and frequency, from climate change, are still dominant in the lower basin [8]. Recent reports from the Sixth phase of Coupled Inter-comparison Project (CMIP6 models), including the Standardized Precipitation Index (SPI) and Standardized Precipitation-Evapotranspiration Index (SPEI) data, predict that seasonal impacts of droughts in the Lower Mekong River Basin (LMRB) will include wetter rainy season and drier non-rainy season conditions. The SPEI shows that the middle reaches of the LMR will experience the highest levels of drought duration and intensity [27].

Reservoirs across the Lancang River reduced the annual average flow by 5% at Chiang Saen during 2008-2016, but their impacts on flow were undetectable downstream at Vientiane Laos. This flow data highlights the importance of site-specific descriptions like that provided by the PBCM since the After Dams data was collected during this period. The streamflow changes noted downstream of Mukdahan in southern Laos, Cambodia, and Vietnam were attributed to local reservoirs and climate change, with the latter changing the magnitude and frequency of floods by up to 14% in Cambodia and 45% in South Vietnam [28]. Current data on annual surface water flow in the LMR indicate a general downward trend during 1960-2010, with no clear trend after 2010. However, while most studies indicate a decreasing trend, a few studies reported the opposite with increasing flow; this may be the result of different data and methods applied in each study [28].

Hydropower development, irrigation expansion, and global climate change were examined in a study of the LMR [29]. The authors suggested studies, including transboundary collaboration in order to address sustainable development goals, could help to meet planned sustainability

objectives. Another study [30], using the year 2040 development scenarios, showed that hydropower and irrigation development predicted limited impacts on annual flows compared to the early development period. Predicted climate change can reduce the streamflow in June between years 2040 to 2080 for some scenarios and increase the streamflow in October in the same period. Furthermore, the streamflow showed significant increases in the long-term during September-November and December-February but showed decreases during March-May and June-August. Hydropower decreased the streamflow in the wet season by 7% but increased in the dry season by 29%. The combined impacts of all driving factors caused substantial flow reductions during the first half of the wet season by - 42%, but increased the flow in the dry season by 40% and 14% compared to the early development and current scenario, respectively [31].

A recent study [32] described an integrated approach with mixed methods and assessed future sustainability challenges in social, hydrological, and ecological dimensions using a case study from the Lower Mekong basin. The study area included the combined basin of the Se Kong, Se San, and Sre Pok (3S) Rivers that deliver approximately 20% of the flow to the Mekong River system. The results suggested that future river flows in the 3S river system could move closer to natural conditions during the dry season, with increased floods during the wet season, and require a shift in the current dam operations, from maintaining minimum flows to reducing flood hazards [32].

Potential impacts specific to *N. aperta/S. mekongi* habitat

The projected changes in flow in the Lancang- Mekong continuum will lead to major impacts on sediment transport and characteristics along different river channel areas and dam reservoir areas. The changes are both local and site-specific. The proposed PBCM framework can help characterize major determinants of the sediment architecture associated with the observed favorable habitat for *N. aperta* (Figs. 3-5). Snail habitat sites along the lower Mekong River can be described as patchy or mosaics habitats that display high intra-and inter-site variability in substrate type, nutrient availability, and primary producer organisms that regulate food sources and water quality [33]. This was evident in the 2008-2010 studies completed by [2,3].

Sediment architecture and water quality are major determinants of the aquatic food web components supporting *N. aperta* populations, particularly macrophytes, microorganisms, and macroinvertebrates. The habitat for *N. aperta* on Khong Island described in the PBCM is conditioned by sediment architecture and particle size distribution. Sediment is a strong determinant of nutrient type, quantity, and availability to support the primary producer base of the food web. Sediments and other substrates (4) are an important source of food and attachment for snails including *N. aperta* (Figs.3-6). Muntz [34] studied the decomposition of organic matter above and below dams in the USA. The results can be used to better understand dam impacts using the PBCM. Muntz found decomposition rates were significantly greater in the impoundments than in one free-flowing section of the river but did not find a significant relationship between decomposition and oxygen or temperature. The average temperature was slightly warmer in the impoundments, suggesting a stronger effect of temperature than oxygen on decomposition.

Current studies in China indicate similar general results with pre- and post-impoundment studies of hydrological impacts on sediment transport in the Han River Basin in China that produce a significant quantity of sediment loss downstream [35]. Sediment descriptions in the PCBM indicate a general decrease in sediment in the Khong Island, Laos area compared to upstream at Ban Dan, Thailand. Estimates of sediment transport in the Lancang-Mekong River system are highly variable but reflect conditions prevailing prior to the ongoing construction of a cascade of seven dams on the Lancang, that will trap an estimated 83% of the Lancang basin sediment when complete [36]. Thus, almost half of the total natural sediment load of the Mekong will be lost in the reservoirs of the Lancang, and the sediment load of the Lower Mekong River will consist largely of sediment derived from sources within the Lower Mekong River basin itself. These sediment losses could lead to site-specific spates of hungry water [37] in areas described in the PBCM causing habitat changes for *N. aperta* due to scouring and bank and channel erosion [38]. The important role of paleogeography and paleoclimate on speciation and endemism in isolated populations of Schistosoma vectors of the genus *Bulinus* in Africa has been described [39]. Snail vectors can be sensitive to changes in the ecological communities in which they are embedded. Macrophyte communities provide attachment substrate and nutrients for snails in littoral habitats of the LMR as described in the PBCM (Figs.5-6). Hydro-morphological alterations influenced the functional diversity and composition of macrophyte communities in the Danube River [40]. Macrophytes were reported to modulate the detritus cycle and change the nutrient supply in *S. mansoni* snail vector habitats [41]. Qualitative and quantitative changes in nutrients can result in significant changes in habitat structure. The enhanced release, export, and transport of diffuse nutrients from watershed forest litter in the

Lancang River basin, during climate change, have been reported using remote sensing data, experimental parameters, and the SWAT model [42].

Tomczyk [43] compared the points closest to and downstream of a Hydro Powerplant (HP) in Poland and noted an increase in concentrations of most heavy metals. Although data on heavy metal contaminants were not included in the PBCM, future studies should include habitat contamination in the areas with water quality degradation. Fine-grained fractions dominate the clays and sandy clays upstream of the hydropower plants, while sands, sandy clay loams, or sandy clays are dominated downstream. The dominance of fine-grained fractions upstream of the HPs favors the accumulation of metals due to the high sorption capacity of these fractions [43]. When comparing the analyzed groups of points, the average pH values are arranged in the following sequence: upstream HP (U) > downstream HP (D). EC has opposite values than pH (i.e., U < D) EC is greater downstream. In the 1972 pre-impoundment studies, the same trend in pH and EC was noted between Ban Dan and Khong Island. The Tomcyzk study may support sustainable sediment management options for use with the PBCM and allows for the development of recommendations related to the rational management of HPs.

Shi (44) developed a niche model using machine learning algorithms to predict the probability of suitable snail habitats for the *S. japonicum* vector *Oncomelania hupensis* in China. The six greatest contributors to predicting potential *O. hupensis* distribution included silt content (13.13%), clay content (10.21%), population density (8.16%), annual accumulated temperatures of ≥0°C (8.12%), night-time lights (7.67%), and average annual precipitation (7.23%). The Before Dams description in the PBCM shows somewhat similar trends in habitat for *N. aperta* regarding the percent sand, silt, and clay at Ban Dan and Khong Island (Fig 3).

Reports describing the Lower Mekong Basin indicate that overall water quality was good up into the 2000s [1,4]. Recent studies indicate that localized degradation has occurred over the past decade near Vientiane City; the Sekong, Sesan, and Srepok Rivers, the Tonle Sap Lake system, and the Mekong Delta [45,46]. Water quality degradation likely corresponds to flow alteration, erosion, sediment trapping, and point and nonpoint wastewater. Habitat updates in the PBCM can be clarified using this type of data at specific sites.

A study of the relationship between climatic, hydrological, and water quality parameters of the LMR flowing through four different countries (Thailand, Cambodia, Lao PDR, and Vietnam) indicated that DO, pH, conductivity, Ca, Mg, Na, K, alkalinity, Cl, $SO4^{2}$– and Si had fair to strong negative correlations with all hydrological parameters. TSS, alkalinity, and conductivity were proposed as sensitive water quality parameters for monitoring the impacts of changing climate in the lower Mekong River [47]. Descriptions of these water quality parameters in the Before and After Dams information in the PBCM can be useful in future monitoring programs.

Current reports describing the overall mainstream water quality and ion analyses in the Lower Mekong basin near Khong Island cite good to excellent conditions like the monsoon cycle and diel cycle data reported in the PBCM Before Dams studies of 1972-73 (Figures 1-10) [1,4]; however, site-specific data were not collected as part of these surveys and other reports [45,46] cite water quality and habitat deterioration at some sites near Khong Island. A river study in France reported that dams did not disturb the seasonality of major anions but did modify silica and phosphorus concentration-flow relationships, especially during low flow. Such changes in the dynamics of river-water composition may affect downstream biological communities [48] and may occur in the LMR.

Three types of Green House Gases (GHGs), carbon dioxide, methane, and nitrous oxide emitted from reservoirs have an important but often ignored impact on climate change and need to be studied in detail in the LMRB. The biogeochemical cycle of carbon in the aquatic environment is understood to be the main mechanism for generating reservoir-based GHG emissions [49]. Future updates of the PBCM should include current data on GHGs.

The impact of temperature change on infections of *S. japonicum* in China was studied using a growing degree-days simulation model. The results predicted changes in transmission patterns and altered frequency and transmission dynamics of *S. japonicum* with climate change [50].

Kalinda [51] used a synthesis of 19 published articles and indicated that temperature rise may alter the distribution, and optimal conditions for breeding, growth, and survival of intermediate host snails, which may eventually increase the spread and/or transmission of schistosomiasis. Other studies in rivers and lakes have reported that transmission may be high at 15-19 ^{o}C. and 20-25°C. respectively [52]. Both temperature effects studies can be used to enhance the habitat descriptions over time in the PBCM. Liu and Xu [53] studied a unique form of flow regulation (e-flow) called hydropeaking that introduces frequent, short-duration, artificial flow events to river systems in China. Hydropeaking is one form of the sporadic flows affecting habitat structure in the After Dams effects noted in the PBCM. They reported that hydropeaking drove plant species distribution, assemblage structure, and species richness-biomass relationships. Hydropeaking also generated complex species-specific effects on plant biomass patterns. They concluded that hydropeaking is likely a major driver of plant community assembly in hydropeaking-affected rivers.

Twenty sites were sampled along the length of the Mekong River from northern Thailand to Lao PDR, Cambodia, and southern Vietnam. Sites included lower Mekong River shorelines and major tributaries. Data were collected on four groups of organisms considered suitable for biomonitoring studies: benthic diatoms, zooplankton, littoral macroinvertebrates, and benthic macroinvertebrates [5,6]. Data from selected lower Mekong River sites in Laos, Thailand, and Cambodia are included in the PBCM, and results indicate that all groups were diverse and abundant in the Mekong River system with considerable variability in abundance and composition from site to site, with the mosaic conditions as described in [15,33]. In another study, thirty-two sites were assessed and classified as to their 'ecological health' using indicator characteristics, including physical and chemical measures, diatoms, and littoral macroinvertebrates. These sites were then classified as excellent, good, moderate, or poor. Ten sites were moderate and one site was poor, with the remaining sites classified as good to excellent [6].

Limpanont et al. [54] reported a new habitat site for the γ- strain of *N. aperta* (with molecular confirmation of identity) from Nong Khai Province, where it occurred in a habitat novel for this species - under paving slabs instead of under natural bed rocks as noted in [4] and PBCM data descriptions. The new site had agal aufwuchs and was located on the islet in the middle of the Mekong River. The new location is approximately four hundred km upstream from the nearest previously known site for this species.

Discussion

Suggested variables and habitat models that can be used along with the PBCM are currently available or under development. Water management and economic development plans should fully consider e-flow recommendations that include specific criteria linked to ecological functions and processes [55,56]. Some of those functions and processes are available using descriptions from the PBCM. A robust approach that includes specific combinations of biological, physical, and chemical data could provide a useful tool to help manage river flows along different impounded areas of the Lancang-Mekong River. Assessments of e-flow should be appropriate for the context and as simple as possible. E-flow regimes should be spatially and temporally specific, connected to ecological processes and functions, and adaptive to changing conditions. One good way of accomplishing this is to develop habitat models that incorporate desired e-flow regime characteristics in the model design. Yarnell and Thoms [57] propose the application of Functional Flows using a flow-chain model and provide two case study examples from Australia and the United States, where improvements in channel habitat and reconnection with the floodplain help achieve the desired functionality of environmental flows. A recent case study reported the use of e-flow models coupled with e-flow allocations for a tropical reservoir system by integration of water quantity (SWAT) and quality (GEFC, QUAL2K) models [58]. The coupled models allowed control of DO and other important water quality parameters that could influence snail habitat and distribution. Coupled e-flow- ecological niche habitat models could be used with information from the PBCM to provide a powerful tool for estimating the potential habitat areas that exist in the highly diverse shoreline mosaic of the Lancang-Mekong watershed. It may be possible to create predictive models for future use by describing suitable habitats for *N. aperta* and other *Schistosoma sp*. snail intermediate hosts along shoreline areas

of the Lancang- Mekong River continuum. These models could be powerful management tools that combine e-flow and other habitat models describing water quality with schistosome biomarkers currently under development [59] in a One Health context to predict possible areas suitable for disease transmission.

Hydro-ecological modeling in SRH-2D and CASiMiR, utilizing flow velocity measurements and macroinvertebrate sampling data, can be considered as one method to design flow regimes that protect biological communities and should include hydrological data reflecting human influences and climate and their effects on sediment distribution [60,61].

An example of a coupled e-flow-habitat model for use in hydraulic schistosomiasis control could combine the ecological niche model as described by Shi [44], with a new model for predicting the terminal settling velocity and drag coefficient of *Oncomelania* [62]. That model could then be integrated with observations from the PBCM and current e-flow studies and habitat models [63,64,65]. Another approach could involve coupling habitat suitability models (HSMs) with frameworks for e-flow estimation models. Szalkiewicz [66] reported a comparative assessment of e-flow values for hydrobiid snails and other macroinvertebrates at two reaches of a river with different levels of hydro morphological change. They noted strong dependence of e-flows on morphological conditions and that the lower limits of e-flows noted were greater than values based on hydrology. Coupling the HSM model with data from the PBCM, including information from data-poor river systems, [16] could enhance the value of both approaches and provide a useful predictive tool for describing ecological habitats in rivers with limited data.

Conclusions

Value and potential applications of the PBCM framework

The PBCM model can serve as a template to summarize, compare, and combine other models describing the habitat-supporting transmission of S. *mekongi* transmission by *N. aperta*. The models can help to select/predict which shoreline areas along the Mekong River could support *N. aperta* and other vector snails, as well as habitats that will support and protect the biodiversity of the river molluscan community in general. Niche models and habitat suitability models can be powerful tools when combined with current e-flow models. Together, the models can help to identify effective e-flow strategies to help with the control and management of waterborne disease transmission. Luo [67] completed a recent systematic review and geospatial analysis of schistosomiasis in Southeast Asia and reported that although the prevalence has been substantially reduced since 2000, focal areas with high prevalence remain. They identified areas, including Khong Island, Laos, that should be targeted for interventions to reduce disease. Models could be built using general descriptions of the littoral zone in [6,15]. PBCM models of this type have been described and suggested for other data-deficient rivers [15,16].

It may be possible to create a suite of predictive models describing suitable habitats for *N. aperta* and other *Schistosoma sp*. that are intermediate hosts along the Lancang- Mekong River continuum shoreline areas. These models could be powerful management tools, in a One Health context, by combining e-flow models with key water quality parameters and schistosome biomarkers currently under development. A Maximum Entropy (Maxent) model predicts suitable habitats for *S. haematobium* and *S. mansoni* in Africa and is a robust snail

suitability model useful in developing control and management programs for schistosomiasis in the study area [68]. Maxent models differ from the PBCM described here by requiring larger sample sizes with a minimum of 10 sampling points for reliability [69] and by requiring presence-only data [70]. Unlike the PBCM, the Maxent model only describes sites where snails are present and cannot be used to predict potential future sites capable of supporting snail intermediate hosts. *S. haematobium* and *S. mansoni* intermediate hosts are amphibian snails with a different niche than the totally aquatic *N. aperta,* and the PBCM, using both presence and absence data describing suitable habitat, may prove to be as robust and useful for sites with limited data. A Bayesian network model (sBN) delineated exposure areas of *S. japonicum* and reports high values of probability of exposure which corresponds to polygons where snails could potentially be present. The PBCM could be the basis to develop a similar model for *S. mekongi.* The schistosomiasis exposure sBN could be used by local disease control teams to identify areas of exposure and improve the efficiency of mass drug administration [71]. A study to address how snail hosts and their interaction with *S. mansoni* influence model predictions indicates that model outputs, such as schistosome prevalence in human and snail populations, respond to the inclusion of snail age structure [72]. Another study linked quantitatively hydrological drivers to distinct population dynamics through specific density feedbacks and shows that model averaging based on statistical methods yields reliable projections of snail abundance [73]. The importance of hydrological drivers of habitat structure is evident in the PBCM and snail habitat models in general. A recent study of projected changes in annual extreme rainfall, and high and low streamflow events over Southeast Asia, under extreme climate change, indicates dramatic fluctuations as predicted by a High-resolution Model

Intercomparison Project (HighResMIP) multi-model experiment for the period 1971-2050 [74]. Some of the model predictions correspond with areas near the 1972 studies in Laos described in this paper and could influence future habitat dynamics of *N. aperta/S. mekongi.*

Acknowledgments

We wish to thank two anonymous reviews for helpful comments, the Interlibrary Loan Staff at the University of Massachusetts, Amherst (ILLiad) for help in accessing information, and Moira Clingman for preparing the graphics.

References

1. Commission (MRC) Mekong River Commission (MRC) [Internet]. www.mrcmekong.org.
2. Dao HG, Kunpradid T, Vongsombath C, Do TBL, Prum S. Report on the 2008 biomonitoring survey of the Lower Mekong River and selected tributaries [Internet]. Mekong River Commission. Vientiane: Mekong River Commission; 2010 Feb [cited 2021 Apr] p. 69 pp. Available from: https://mrcmekong.org
3. Davidson SP, Kunpradid T, Peerapornisal Y, Nguyen TML, Pathoumthong B, Vongsambath C, Biomonitoring of the lower Mekong River and selected tributaries. MRC Technical Paper No 13, Mekong River Commission, Vientiane; 2006. 100 pp; ISSN: 1683-1489.
4. Kitikoon V, Schneider C, Sornmani S, Harinasuta C, Lanza G. Evidence of the natural transmission of *Schistosoma japonicum,* Mekong strain, at Khong Island Laos. Southeast Asian Journal of Tropical Medicine. 1973 Sep(43):350–8. PMID:4768712.

5. Lanza GR. Eutrophication: causes, consequences, and control [Internet]. Ansari AA, Gill SS, Lanza GR, Rast W, editors. Dordrecht: Springer; 2011. Available from: https://doi.10.1007/978-94-007-7814-6-9625-8 2

6. Sor R, Ngor PB, Boets P, Goethals PLM, Lek S, Hogan ZS, Park Y-S. Patterns of Mekong mollusc biodiversity: identification of emerging threats and importance to management and livelihoods in region of globally significant biodiversity and endemism. Water 2020,12(9) 2619. https://doi.org3390/w12092619

7. Wang W, Lu H, Ruby Leung L, Li H-Y, Zhao J, Tian F, et al. Dam construction in Lancang-Mekong River Basin could mitigate future flood risk from warming-induced intensified rainfall. Geophysical Research Letters. 2017 Oct 28;44(20):10-378. https://doi.org/10.1002/2017GLO75037

8. Khir Alla YM, Liu L. Impacts of dams on the environment: a review. International Journal of Environment, Agriculture and Biotechnology. 2021;6(1):064–74.

9. Dong Z, Liu H, Baiyinbaoligao, Hu H, Khan MYA, Wen J, et al. Future projection of seasonal drought characteristics using CMIP6 in the Lancang-Mekong River Basin.

10. Yun, Xiaobo, et al. Impacts of climate change and reservoir operation on streamflow and flood characteristics in the Lancang-Mekong River Basin. Journal of Hydrology. 202 Nov. 1;590:125472 https://doi.org/10.1016/j.jhydrol.2020.125472

11. Junk W, Blayey P, Sparks R. The flood pulse concept in river-floodplain systems. Canadian Special Publication Fisheries Aquatic Sciences 106; 1989 p. 10pp.

12. Poff NL, Allan JD, Bain MB, Karr JR, Prestegaard KL, Richter BD, et al. The natural flow regime. BioScience. 1997 Dec;47(11):769–84. https://doi.org/10.2307/1313099

13. Fan H, He D, Wang H. Environmental consequences of damming the mainstream Lancang-Mekong River: A review. Earth-Science Reviews. 2015 Jul; 146:77–91. https://doi.org/10.1016/j.earscirev.2015.03.007

14. Poff NL. A river that flows free connects up in 4D. Nature. 2019 May;569(7755):201–2. https://doi.org/10.1038/d41586-019-01393-2

15. Brierley GJ, Fryirs KA. River futures: an integrative scientific approach to river repair. Washington, DC, Tucson, Ariz.: Island Press; Restor Ecol, 2008

16. Sharma U. Effective framework for environmental-flows estimation for data deficient Indian rivers. Journal of Applied and Natural Science. 2019 Jun 10;11(2):545–55. https://doi.org/10.31018/jans.v11i2.2116

17. Blair D, Xu Z-B, Agatsuma T. Paragonimiasis and the Genus *Paragonimus*. Advances in Parasitology. 1999;113–222.

18. Upatham E. Field studies on the bionomics of alpha and gamma races of *Tricula aperta* in the Mekong River at Khemmarat Ubol Ratchanthani province, Thailand. Malacology Review. 1980;2:239–61.

19. Nishina M. The composition of the Mekong River Silt and its possible role as a source of plant nutrients in the delta [Internet] [MSc]. [University of Hawaii]; 1974 [cited 2022 Mar]. p. 1–109. Available from: https://scholarspace.manoa.hawaii.edu/handle/10125/56378

20. Stauffer, JR, Arnegard ME, Cetron M, Sullivan JJ, Chitsulo LA, Turner GF, et al. Controlling vectors and hosts of parasitic diseases using fishes. BioScience. 1997;47: 41–49. https://doi:10.2307/1313005

21. Riehl R, Allen DJ, Baensch HA. Skunk loach. Wikipedia. Wikimedia Foundation; 2023. Available: https://en.wikipedia.org/wiki/Skunk_loach

22. Tiegtam N, Pongpat C, Sangchan J. Feeding habits of the snail-eating catfish (*Pangasius conchophilus Roberts & Vidthayanon,* 1991) in Mekong River Nakhon Pano Province. Proceedings of the 48th Kasetsart University Annual Conference, Kasetsart, 3-5 March 2010 Subject: Fisheries. Kasetsart, Thailand: Kasetsart University.

23. Knudson HP, Curran SS, Truong TN, Dutton HR, Bullard SA. *Encyclobrephus brevivitellus* n. sp. (Digenea: *Plagiorchioidea, incertae sedis*) infecting the Mekong snail-eating turtle, *Malayemys subtrijuga* , in the Mekong River, Vietnam, with phylogenetic analyses based on rdna. Journal of Parasitology. 2023;109: 87–95. https://doi:10.1645/22-98

24. Shimada M, Kato-Hayashi N, Chigusa Y, Nakamura S, Ohmae H, Sinuon M, et al. High susceptibility of *Neotricula aperta* gamma-strain from Krakor and Sdau in Cambodia to *Schistosoma mekongi* from Khong Island in Laos. Parasitology International. 2007 Jun;56(2):157–60.

25. Institut Pasteur Du Laos Activities Report 2017-2018 : Institut Pasteur Du Laos. www.pasteur.la, 2018, www.pasteur.la/annual-report/institut-pasteur-du-laos-activities-report-2017-2018/.

26. Kumagai T, Matsumoto-Takahashi ELA, Ishikawa H, Keomalaphet S, Khattignavong P, Soundala P, et al. Detection of *Schistosoma mekongi* DNA in human stool and intermediate host snail *Neotricula aperta* via loop-mediated isothermal amplification assay in Lao PDR. Pathogens. 2022 Nov 24;11(12):1413. https://doi.10.3390/pathogens11121413

27. Dong Z, Liu H, Baiyinbaoligao, Hu H, Khan MYA, Wen J, et al. Future projection of seasonal drought characteristics using CMIP6 in the Lancang-Mekong River Basin. Journal of Hydrology. 2022 Apr;127815. https://doi.org/10.1016/j.jhydrol.2022.127815

28. Yun X, Tang Q. Wang J, Liu X, Zhang Y, Lu H. et al. Impacts of climate change and reservoir operation on streamflow and flood characteristics in the Lancang-Mekong River Basin. Journal Hydrology, of vol. 590, Nov. 2020, p. 125472, https://doi.org/10.1016/j.jhydrol.2020.125472

29. Savathdy P, Kinouchi T, Khanophet P. Analysis of water infrastructure development and climate change impact on the streamflow in the lower Mekong River basin. Journal of Japan Society of Civil Engineers, Ser B1 (Hydraulic Engineering). 2021;77(2):I265–70.

30. Lauri H, de Moel H, Ward PJ, Räsänen TA, Keskinen M, Kummu M. Future changes in Mekong River hydrology: impact of climate change and reservoir operation on discharge. Hydrology and Earth System Sciences. 2012 Dec 5;16(12):4603–19

31. Liu J, Chen D, Mao G, Irannezhad M, Pokhrel Y. Past and future changes in climate and water resources in the Lancang–Mekong River Basin: current understanding and future research directions. Engineering, Sept. 2021, https://doi.org/10.1016/j.eng.2021.06.026. Accessed 12 Oct. 2021.

32. Mohammed I, Bolten J, Souter N. Shaad K, Vollmer D. Assessment of water resources conservation and sustainable management strategies in the Lower Mekong River Basin. 27 Mar. 2022, https://doi.org/10.5194/egusphere-egu22-1323

33. Cullum C, Brierley G, Thomas M. The spatial organization of river systems. In: Brierley G, Fryirs K, editors. River futures an integrative scientific approach to river repair. Island Press; 2008.

34. Muntz O. Decomposition of organic matter above and below dams [Internet]. [Eastern Michigan University]; 2022 [cited 2022]. Available from: https://commons.emich.edu/honors/726

35. Jiang, Chong, Understanding the hydropower exploitation's hydrological impacts through a lens of change in flow-sediment relationship: a case study in the Han River Basin, China. Ecological Engineering. 2019 Apr 1;129:82–96. https://doi.org/10.1016/j.ecoleng

36. Kondolf, GM, Rubin ZK, Minear JT. Dams on the Mekong: Cumulative Sediment Starvation. Water Resources Research. 2014 June;50(6):5158–5169. https://doi.org/10.1002/2013wr014651.

37. Kondolf GM. PROFILE: Hungry water: effects of dams and gravel mining on river channels.   Environmental Management [Internet]. 1997 Jul 1;21(4):533–51. Available from: https://people.wou.edu/~taylors/g407/kondolf_97.pdf

38. Rubin ZK, Kondolf GM, Carling PA. Anticipated geomorphic impacts from Mekong Basin Dam construction. International Journal of River Basin Management, 2015 Jan 2;13(1):104-21. https://doi.org/10.1080/15715124.2014.981193.

39. Tumwebaze I, Clewing C, Chibwana FD, Kipyegon JK, Albrecht C. Evolution and biogeography of freshwater snails of the Genus *Bulinus* (Gastropoda) in Afromontane

extreme environments. Frontiers in Environmental Science. 2022:572. https://doi.org/10.3389/fenvs.2022.902900. Accessed 6 Dec. 2022.

40. Vukov D, Ilic M, Cuk M, Igic R. The effect of hydro-morphology and habitat alterations on the functional diversity and composition of macrophyte communities in the large river." Frontiers in Environmental Science, 2022:377. https://doi.org/10.3389/fenvs.2022.863508. Accessed 6 Dec. 2022.

41. Desautels DJ, Hartman RB, Shaw K, Maduraiveeran S, Civitello DJ. Divergent effects of invasive macrophytes on population dynamics of a snail intermediate host of *Schistosoma Mansoni.* Acta Tropica. 2022 Jan;225:106226.

42. Hao H, Ouyang W, Zhang K, Wan X, Cui X, Zhu W. Enhanced release, export, and transport of diffuse nutrients from litter in forested watersheds with climate warming." Science of the Total Environment, 2022 Sep 1:837:155897. https://doi.org/10.1016/j.scitotenv.2022.155897. Accessed 7 Dec. 2022.

43. Tomczyk P, Wiatkowski M, Galka B, Gruss L. Assessing the impact of a hydropower plant on changes in the properties of the sediment of the Bystrzyca River in Poland. Frontiers in Environmental Science. 2022;10. https://doi.org/10.3389/fenvs.2022.795922. Accessed 7 Dec. 2022.

44. Shi Q, Gong Y, Zhao J, Qin Z, Zhang J, Wu J et al. Spatial and temporal distribution pattern of *Oncomelania Hupensis* caused by multiple environmental factors using ecological niche models. Frontiers in Environmental Science. 2022:888. https://doi.org/10.3389/fenvs.2022.942183. Accessed 3 Dec. 2022.

45. Sor R, Ngor PB, Soum S, Chandra S, Hogan ZS, Null SE. Water Quality Degradation in the Lower Mekong Basin.Water. 2021 May 31;13(11):1555. https://doi.org/10.3390/w13111555. Accessed 20 Dec. 2021.

46. Jutmas S, Kwansiri R, Vongsombath C, Viengkhone V, Chutima H, Narumon S. Benthic Macroinvertebrates and Trichoptera adults for bioassessment approach in streams and wadeable rivers in Lao People's Democratic Republic. Tropical Natural History [Internet]. 2022;22:12–24. Available from: https://li01.tci-thaijo.org/index.php/tnh/article/view/254817

47. Prathumratana L, Sthiannopkao S, Kim KW. The relationship of climatic and hydrological parameters to surface water quality in the lower Mekong River. Environment International. 2008 Aug;34(6):860–6.

48. Fovet O, Ndom M, Crave A, Pannard A. Influence of dams on river water-quality signatures at event and seasonal scales: The Sélune River (France) Case Study. River Research and Applications. 2000 Sep 36(7):1267-78. https://doi.org/10.1002/rra.3618.

49. Lu S, Dai W, Tang Y, Guo M. A review of the impact of hydropower reservoirs on global climate change. Science of The Total Environment. 2020 Apr 1;711:134996.

50. Yang G, Gemperli A, Utzinger J, Zhou X-N, Vounatsou P, Tanner M. A growing degree-days based time-series analysis for prediction of *Schistosoma japonicum* transmission in Jiangsu province, China. The American Journal of Tropical Medicine and Hygiene. 2006 Sep 1;75(3): 549–555. doi:10.4269/ajtmh.2006.75.549

51. Kalinda C, Chimbari M, Mukaratirwa S. Implications of changing temperatures on the growth, fecundity and survival of intermediate host snails of Schistosomiasis: A

systematic review. International Journal of Environmental Research and Public Health [Internet]. 2017 Jan 1;14(1). Available from: https://www.ncbi.nlm.nih.gov/pmc/articles/PMC5295331/

52. McCreesh N, Booth M. The effect of increasing water temperatures on *Schistosoma mansoni* transmission and *Biomphalaria pfeifferi* population dynamics: an agent based modelling study. PLOS ONE 2014 Jul 2;(7):e101462. https://doi.org/10.1371/journal.pone.0105917.

53. Liu X and Xu Q. Hydropeaking impacts on riverine plants downstream from the world's largest hydropower dam, the Three Gorges Dam. Science of the Total Environment. 2022 Nov 1;845:157137. https://doi.org/10.1016/j.scitotenv.2022.157137.

54. Limpanont Y, Chusongsang P, Chusongsang Y , Limsomboon J, Sanpool O, Kaewkong W, et al. A new population and habitat for *Neotricula Aperta* in the Mekong River of Northeastern Thailand: A DNA sequence-based phylogenetic assessment confirms identifications and interpopulation relationships. The American Journal of Tropical Medicine and Hygiene. 2015 Feb 2;92(2):336. https://doi.org/10.4269/ajtmh.14-0467

55. International Water Resources Association [Internet] Environmental Flows: Harmony between Humans and Nature. International Water Resources Association Policy Brief 22; 2022 May. Available from: https://www.iwra.org

56. Arthington AH, Kennen JG, Stein ED, Webb JA. Recent advances in environmental flows science and water management-Innovation in the Anthropocene. Freshwater Biology. 2018 Apr 16;63(8):1022–34.

57. Yarnell SM, Thoms M. Enhancing the functionality of environmental flows through an understanding of biophysical processes in the riverine landscape. Frontiers in Environmental Science. 2022 Oct 21;10.

58. Kumar MS, Priya PN, Shaik R, Singh SK. Environmental flows allocation for a tropical reservoir system by integration of water quantity (SWAT) and quality (GEFC, QUAL2K) models. Water Resources Management. 2023 Jan;37(1):113–33. https://doi:10.1007/s11269-022-03358-z

59. Uthailak N, Adisakwattana P, Thiangtrongjit T, Limpanont Y, Chusongsang P, Chusongsang Y, et al. Discovery of *Schistosoma mekongi* circulating proteins and antigens in infected mouse sera. Plos One. 2022 Oct 13;17(10):e0275992.

60. Hough IM, Warren PH, Shucksmith JD. Designing an environmental flow framework for impounded river systems through modelling of invertebrate habitat quality. Ecological Indicators. 2019 Nov 1;106:105445. https://doi.org/10.1016/j.ecolind.2019.105445. Accessed 17 July 2021.

61. Jay DA, Naik PK. Distinguishing human and climate influences on hydrological disturbance processes in the Columbia River, USA. Hydrological Sciences Journal. 2011 Oct 1;56(7):1186-209. https://doi.org/10.1080/02626667.2011.604324. Accessed 10 Mar. 2020.

62. Liu X, Wang J, Chai Z, Min F, Jiang X, Zhu K. et al. New Model for Predicting Terminal Settling Velocity and Drag Coefficient of the *Oncomelania.* Frontiers in Environmental Science. 2022 Nov 2;10:2199. https://doi.org/10.3389/fenvs.2022.1051392. Accessed 8 Dec. 2022.

63. Chen A, Wu M, Chen KQ, Sun ZY, Shen C, Wang PY. et al. Main Issues in research and practice of environmental protection for water conservancy and hydropower projects in China. Water Science and Engineering. 2016 Oct 1;9(4):312-23. https://doi.org/10.1016/j.wse.2017.01.008. Accessed 19 Nov. 2022.

64. Chen A, Wu M, Wu SN, Sui X, Wen JY, Wang PY. Bridging gaps between environmental flows theory and practices in China. Water Science and Engineering. 2019 Dec 1;12(4):284-92. https://doi.org/10.1016/j.wse.2019.12.002. Accessed 8 Dec. 2022.

65. Chen A, Wu M. Managing for sustainability: the development of environmental flows implementation in China. Water. Feb 28;11(3):433. https://doi.org/10.3390/w11030433.

66. Szałkiewicz E, Kaluza T, Grygoruk M. Environmental flows assessment for macroinvertebrates at the river reach scale in different degrees of hydromorphological alteration. Frontiers in Environmental Science. 2022 Apr;11;10:386. https://doi.org/10.3389/fenvs.2022.866526

67. Luo C, Wang Y, Su Q, Tang S, Bergquist R, Zhang Z, Hu Y. Mapping schistosomiasis risk in Southeast Asia: a systematic review and geospatial analysis. International Journal of Epidemiology, 2022; Dec 8:1-13. https://doi.org/1093/ije/dya c 227

68. Manyangadze T, Chimbari MJ, Gebreslasie M, Ceccato P, Mukaratirwa S. Modelling the spatial and seasonal distribution of suitable habitats of schistosomiasis intermediate host snails using Maxent in NDUMO area, KwaZulu-Natal Province, South Africa. Parasites Vectors. 2016 Dec;9(1):572. https://doi.org/10.1186/s13071-016-1834-5.

69. Wisz MS, Hijmans RJ, Li J, Peterson AT, Graham CH, Guisan A. Effects of sample size on the performance of species distribution models. Diversity and Distributions. 2008 Sep;14(5):763–773. https:doi:10.1111/j.1472-4642.2008.00482.x

70. Phillips SJ, Dudík M. Modeling of species distributions with Maxent: New extensions and a comprehensive evaluation. Ecography. 2008 Apr;31(2):161–175. https://doi:10.1111/j.0906-7590.2008.5203.x

71. Araujo Navas AL, Soares Magalhaes RJ, Osei F, Fornillos RJ, Leonardo LR, Stein A. Modelling local areas of exposure to *Schistosoma japonicum* in a limited survey data environment. Parasites & Vectors. 2018 Dec;11(1):1-5.

72. Anderson, L C., Loker ES, Wearing HJ. Modeling schistosomiasis transmission: the importance of snail population structure. Parasites & Vectors. 2021 Dec ;14:1-4. https://doi.org/10.1186/s13071-021-04587-8.

73. Perez-Saez J, Mande T, Ceperley N, Bertuzzo E, Mari L, Gatto M. et al. Hydrology and density feedbacks control the ecology of intermediate hosts of schistosomiasis across habitats in seasonal climates. Proceedings of the National Academy of Sciences. 2016 Jun 7;113(23):6427–32. https://doi.org/10.1073/pnas.1602251113.

74. Hariadi MH, van der Schrier G, Steeneveld GJ, Sutanto S, Sutanudjaja E, Ratri DN, et al. A high-resolution perspective of extreme rainfall and river flow under extreme climate change in Southeast Asia. Hydrology and Earth System Science Discussions. 2023 Jan 16:1-29. http://doi:10.5194/hess-2023-14

Figure

Click here to access/download;Figure;Fig 1. Placed-based Conceptual Model.eps

Before Dams	After Dams	Future
Historical Habitat Descriptions 1971-1973<br><br>1. Shoreline (littoral)<br>2. Offshore (channel current)	Selected Updated Descriptions 2008-2022<br><br>1. Shoreline (littoral)<br>2. Offshore (channel current)	Selection of Potential Habitat Sites for More Detailed Study Using PBCM and Recent Habitat Descriptions and Models
Sediment, Water Quality, Biota Figures 1-5 Tables 1 & 2	Sediment, Water Quality, Biota 9 sites in Laos, Thailand, Cambodia 2008-2010; Other Updates	Impacts of Climate Change and Human Activity
Habitat Influenced by Natural Flow Regime	Habitat Influenced by Impacts of Dams	Habitat Influenced by Management Strategies, E-flow applications
Monsoon Control	Regulated Flow of Water and Sediment from Dam Releases	Develop New Management Strategies

Figure

Click here to access/download;Figure;Fig 2. Map of the study area.eps

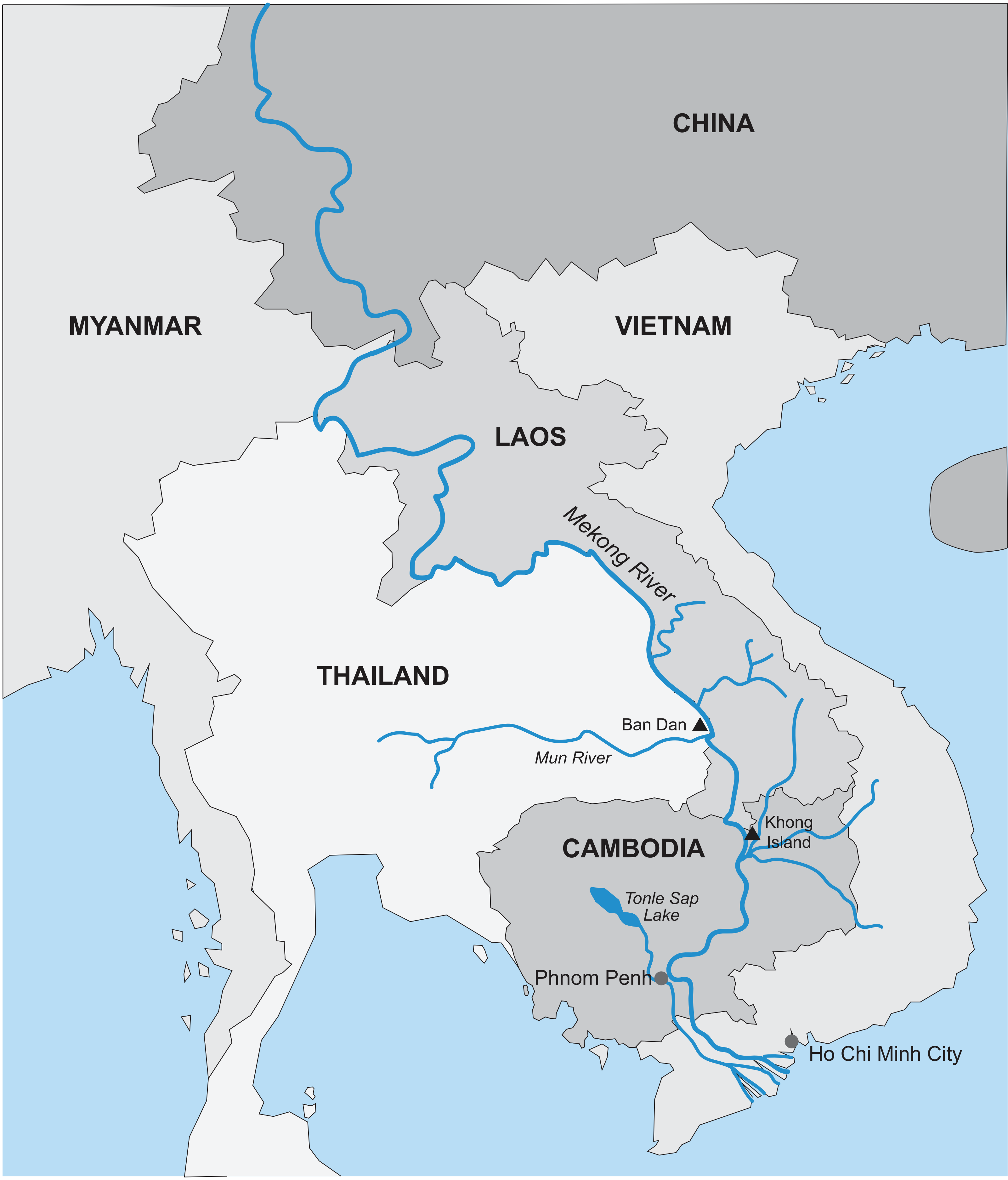

Figure
Click here to access/download;Figure;Fig 3. Habitat description and food web.eps

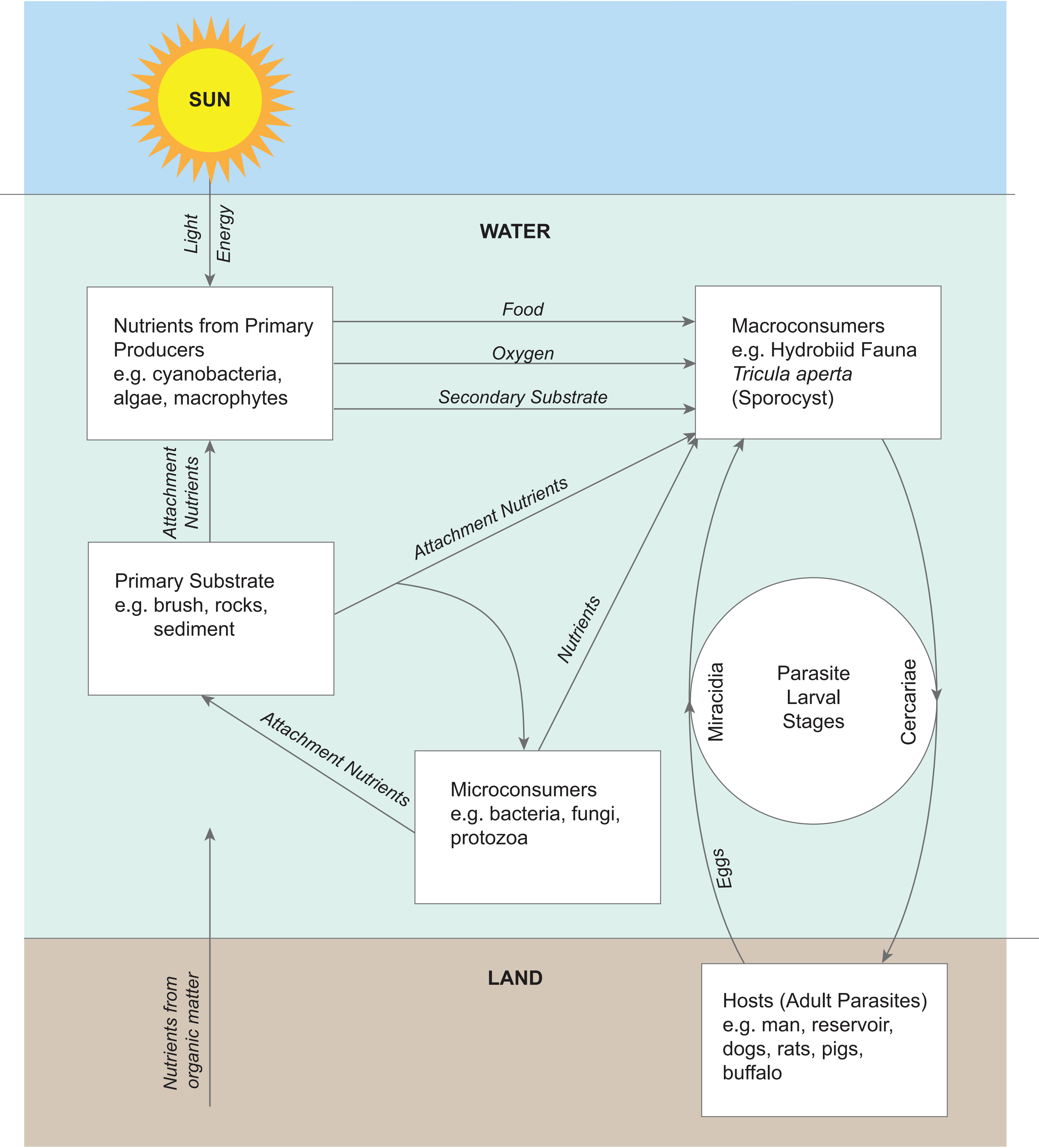

Figure

Click here to access/download;Figure;Fig 4. Water quality one monsoon cycle.eps

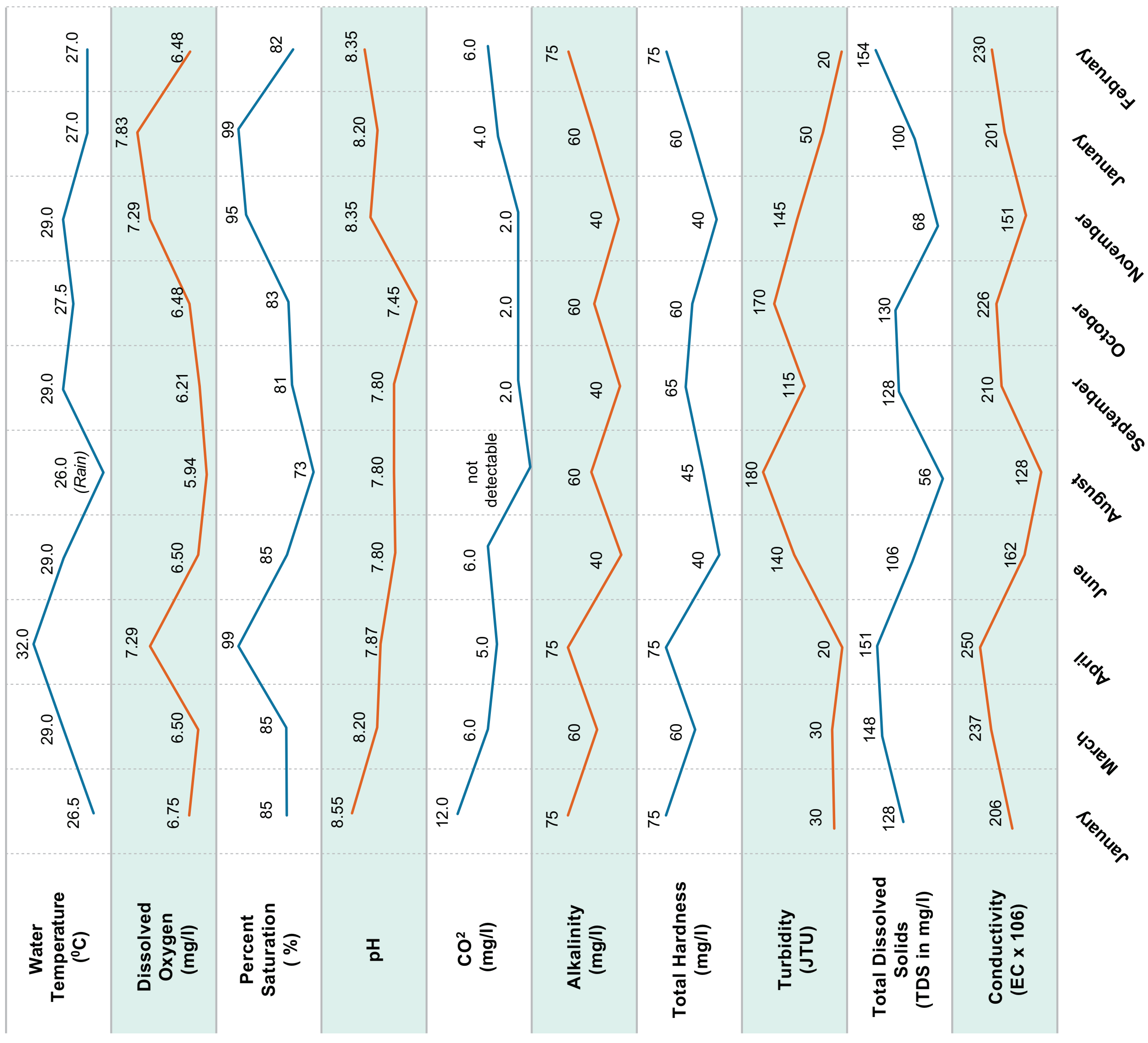

Figure

Click here to access/download;Figure;Fig 5. Water ions one monsoon cycle.eps

Figure 2. Water Ion Analyses in the Mekong River During One Monsoon Cycle in Thailand and Laos 1972-73 (mg/l).

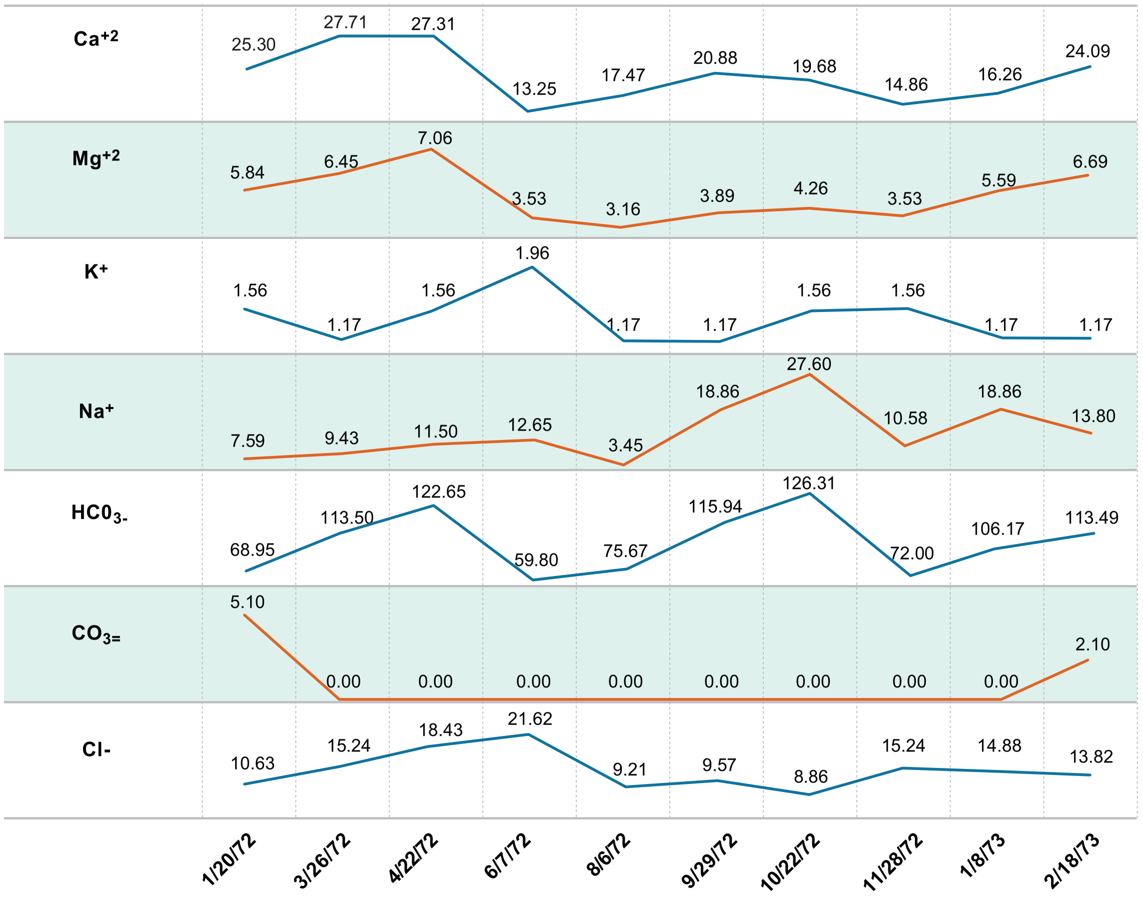

April 21, 1972, Location 1: Area near shoreline with little flow

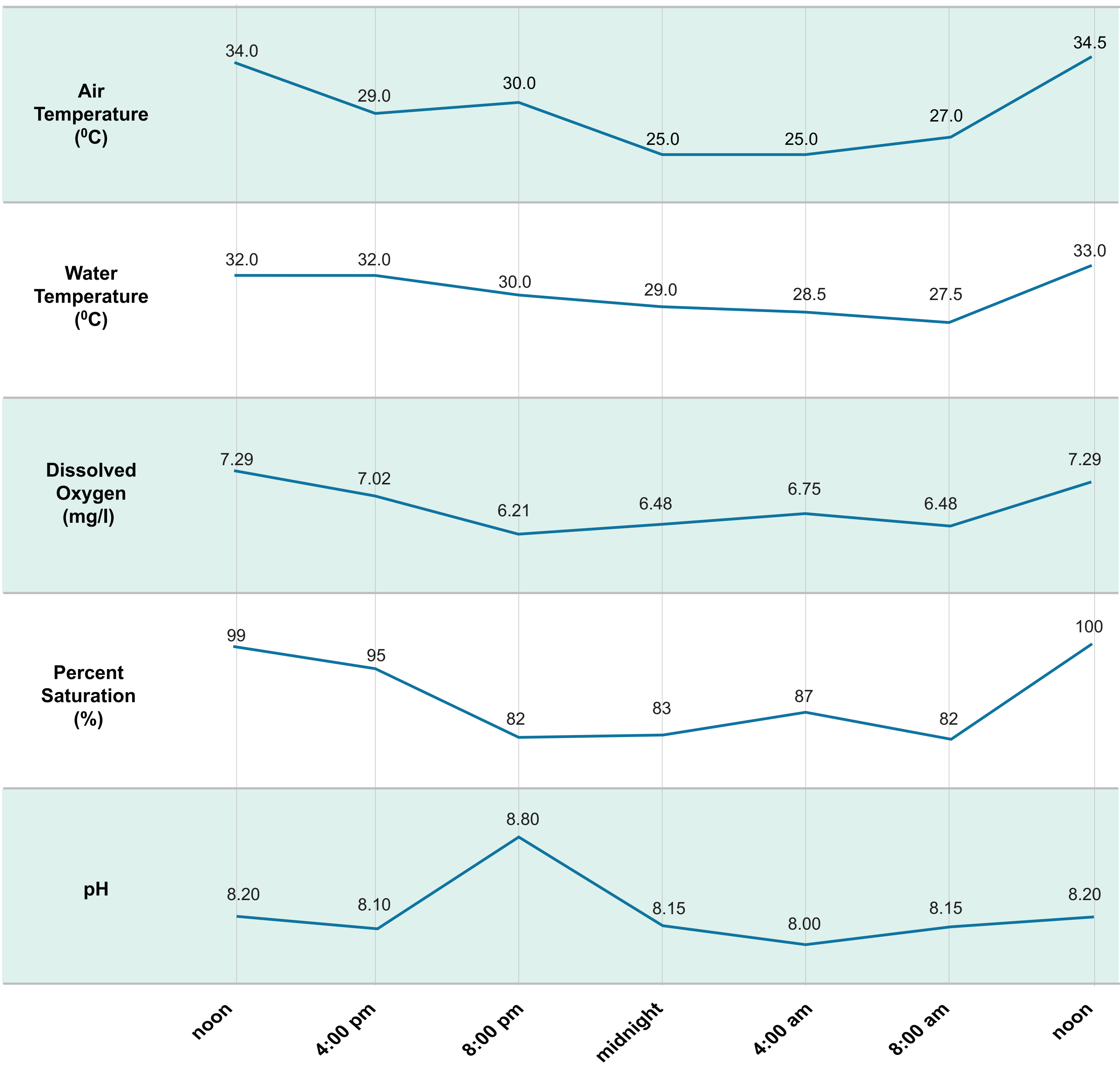

Figure

Click here to access/download;Figure;Fig 6B. 24-hour (diel) cycle April 21, 1972 2.eps

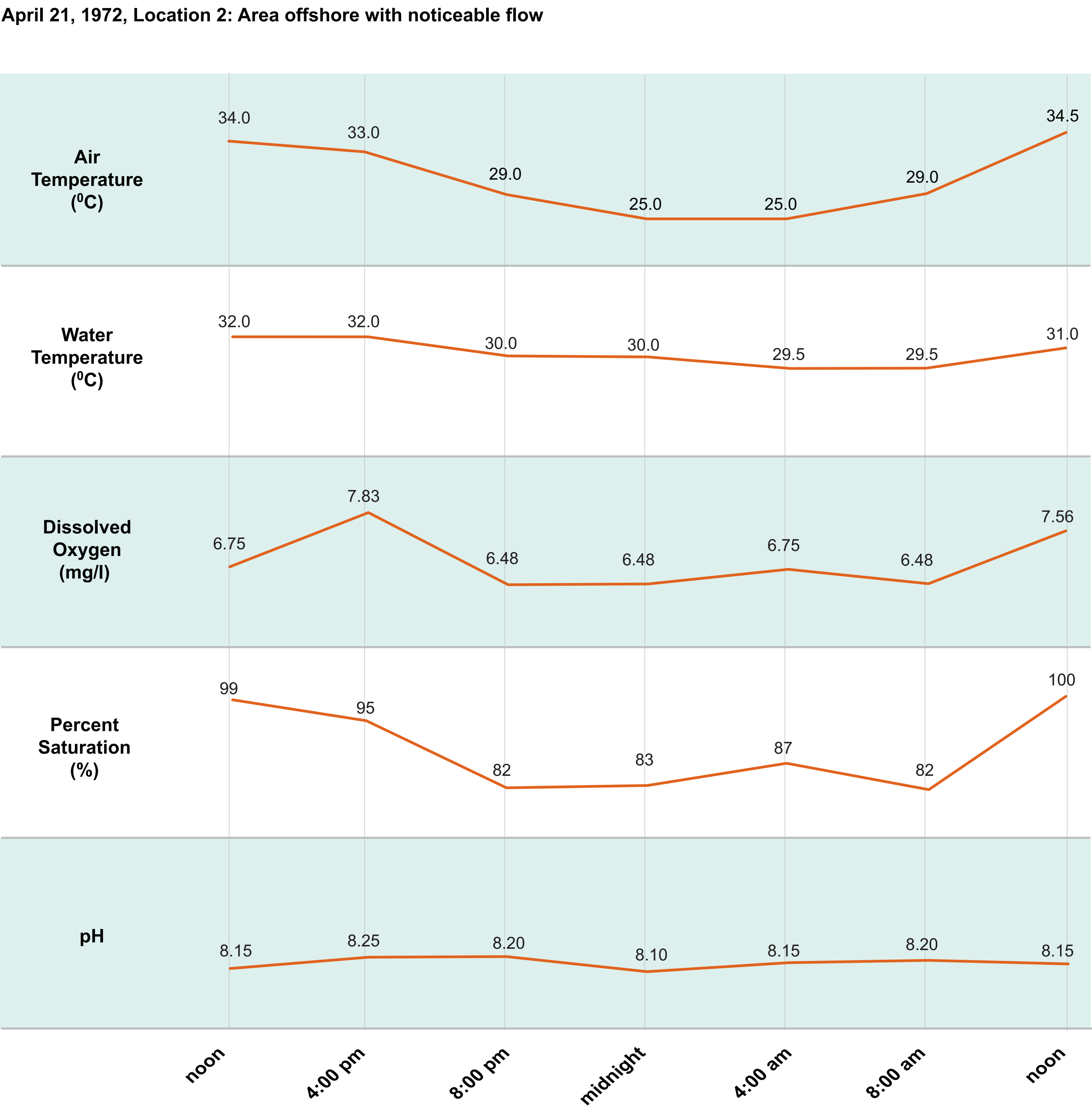

Figure

Click here to access/download;Figure;Fig 7A. 24-hour (diel) cycle April 27, 1972.eps

April 27, 1972, Location 1: Area near shoreline with little flow

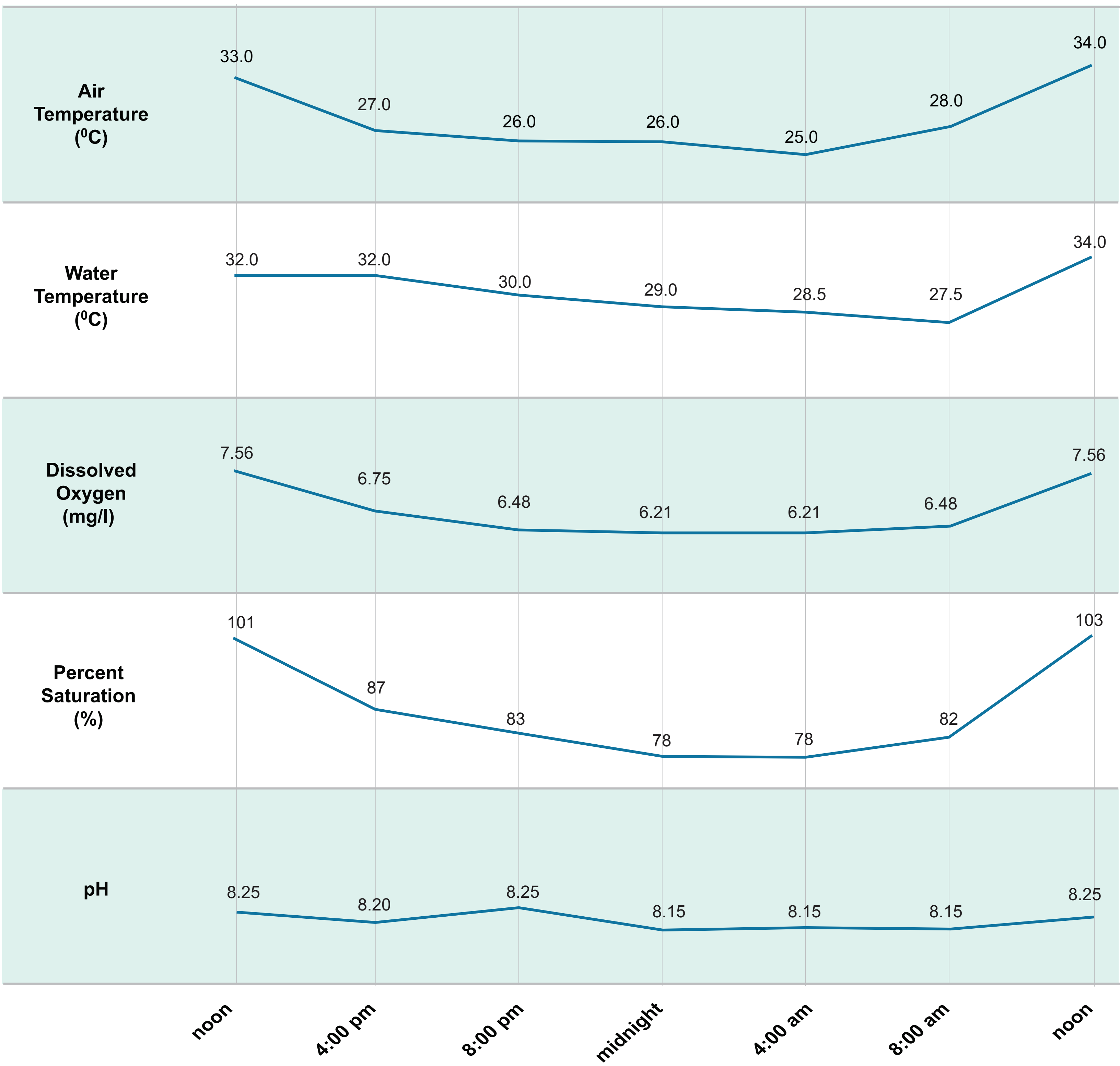

Click here to access/download;Figure;Fig 7B. 24-hour (diel) cycle April 27, 1972.eps

April 27, 1972, Location 2: Area offshore with noticeable flow

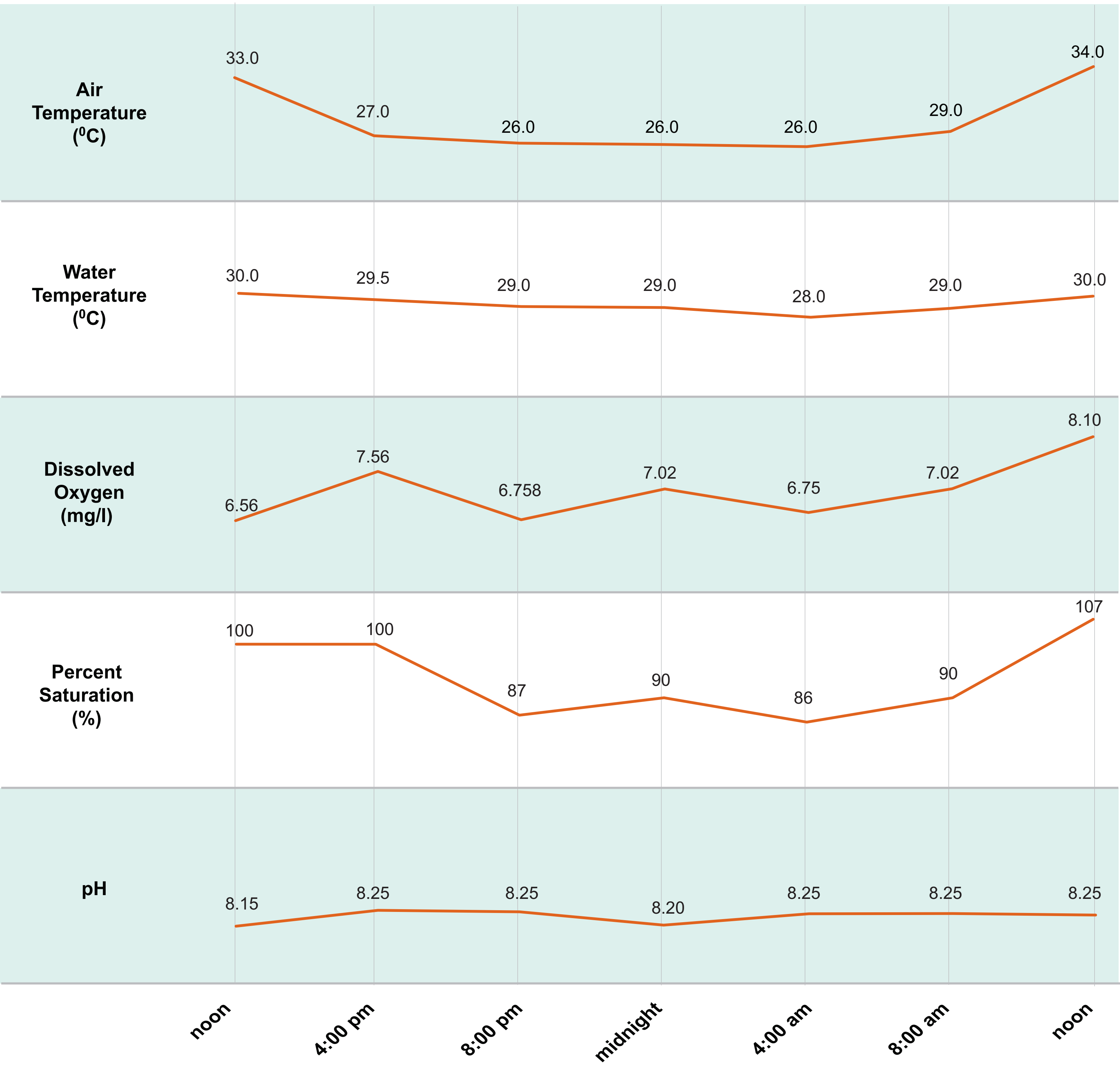

Figure

Click here to access/download;Figure;Fig 8. Sediment characteristics.eps

Particle size (%)[a]	MeB[b]		MeK[c]		
	Mean	S.D.	Mean	S.D.	Significant
Very coarse sand (1.0-2.0)	0.08	0.04	0.05	0.71	
Coarse sand (0.05-1.0)	0.53	0.20	1.33	1.81	
Medium sand (0.25-0.05)	3.20	1.95	1.34	0.85	
Fine sand (0.10-0.25)	29.25	18.80	25.41	10.70	
Very fine sand (0.05-0.10)	16.88	7.79	45.87	7.03	x
Total sand (0.05-2.0)	50.12	27.10	77.94	5.80	
Silt (0.002-0.05)	38.05	21.10	16.36	5.30	
Clay (≤0.002)	11.83	6.08	5.70	3.20	
Organic carbon (%)	0.75	0.33	0.25	0.09	x
Available phosphorus (mg L^{-1})	30.43	3.81	31.2	3.82	
Active iron (%)	1.61	0.58	0.99	0.11	x
Saturation extract ECe@25C (µmho)	0.62	0.07	0.81	0.44	
**Soluable cations (meq $100g^{-1}$)**					
Ca^{2+}	4.93	0.63	5.80	2.43	
Mg^{2+}	1.15	0.13	1.45	0.82	
Na^{+}	0.76	0.13	1.21	0.89	
K^{+}	0.13	0.02	0.33	0.38	
**Soluable anions (meq $100g^{-1}$)**					
Cl^{-}	0.60	0.16	1.00	0.52	
HCO_3^{-}	3.87	0.45	3.39	0.44	
CO_3^{-}	0.00	0.00	0.00	0.00	
**Effective exchangeable cations (meq 100 g^{-1})[d]**					
Ca^{2+}	13.63	6.20	7.12	1.12	x
Mg^{2+}	1.28	0.75	0.86	0.25	
Na^{+}	0.02	0.04	0.01	0.01	
K^{+}	14.93	6.99	8.16	1.2	

a. Bracketed values are particle size ranges in mm

b. MeB, Mekong River at Ban Dan, Thailand *(n=6)*

c. MeK, Mekong River at Khong Island, Laos

d. Extractable cations (1 N KCl) minus soluable cations.

S.D.

Figure
Click here to access/download;Figure;Fig 9. Algal-snail associations.eps

Microhabitat	Algal Assemblage	Gastropods on Algae
Algae attached to wood and rock outcroppings or as free-floating mats 5 meters off shore.	*Ulothrix* sp. with dense epiphytic communities of *Malleodendron* sp. and *Navicula* sp.	*Tricula aperta*<br>*Pachydrobia mcmulleni*
Free-floating macrophytes near shorelines.	*Azolla* sp. with epiphytic *Navicula* sp.	*Stenothyra hybocystoides*<br>*S. basisculpta*
Algae attached to submerged twigs: rocks and leaves near shoreline.	*Spirogyra* sp.<br>*Oedogonium* sp.<br>*Ulothrix* sp. with epiphytic *Malleodendron* sp. Twigs with epiphytic *Spirulina* sp. and *Fragilaria* sp. leaves with epiphytic Closterium sp. and *Navicula* sp.	*T. aperta*<br>*S. hybocystoids*<br>*S. basisculpta*<br>*S. cambodiensis*<br>*P. crooki*<br>*P. wykoffi*<br>*P. mcmulleni*

Figure
Click here to access/download;Figure;Fig 10 Snail species.eps

Table . Distribution Patterns of Littoral ZoneSnail Species, Khong Island, Laos 1972*

Species	Locale**						
	(1)	(2a)	(2b)	(3a)	(3b)	(4a)	(4b)
*Tricula aperta**	+	+	+	+	+	+	+
Stenothyra basisculpta Brant	+	+	+	+	-	+	-
S. hybocystoides Bavay	+	+	+	+	+	+	+
S. laotiensis Temcharoen	-	-	-	-	+	-	-
S. cambodiensis Brandt	-	+	-	-	-	-	-
Stenothyra sp. Juveniles	+	+	-	-	+	+	+
Manningiella expansa Brandt	+	+	-	-	+	+	+
M. pellucida Bavay	+	+	-	-	+	+	+
Pachydrobia mcmulleni[1]	-	+	-	-	-	-	-
P. crooki	-	+	-	-	-	-	-
P. wykoffi	-	+	-	-	-	-	-
Pachydrobiella brevis Bavay	+	+	-	-	+	+	+
Jullienia acuta Poirier	+	-	-	-	-	-	-
J. harmandi Poirier	+	-	-	-	-	-	-
Paraprosothenia iijimai Brandt	+	-	-	-	-	-	-
P. levayi Brandt	-	+	-	-	-	-	-
Lacunopsis fisherpiettei Brandt	-	+	-	-	-	-	+
L. ventricose Poirier	+	-	-	-	-	-	+
Lacunopsis sp. Juveniles	-	+	-	-	-	-	+
Clea (Anentome) helena Philippi	-	+	+	-	-	+	-
Wattebledia crosseana Wittebled	+	-	-	-	-	-	-
Wattebledia sp. Juveniles	-	-	-	-	-	+	-
Hydrorissoia hospitalis Brandt	+	-	-	-	-	-	-
Bithynia sp. Juveniles	-	-	-	-	-	-	+

* *Lithoglyphopsis aperta* Temcharoen, vector of human Schistosomiasis

** Numbers in brackets identify east shore locale; a and b are stations collected within a locale.
+ and – represent present or absent respectively. See F-4 Kitikoon et al. 1973
(1) Military camp area
(2) Ban Xieng Wang, site of documented Schistosomiasis transmission
(3) Dooley Hospital area
(4) Ban Na area

April 9, 2023

Professor Krystyna Cwiklinski
Academic Editor
PLOS Neglected Tropical Diseases

Professor Alvaro Acosta-Serrano
Section Editor,
PLOS Neglected Tropical Diseases

Dear Professors Cwiklinski and Acosta-Serrano,

We have revised PNTD-D-23-00131(1)jrs.pdf using the helpful suggestions of the reviewers to clarify and improve the text. The revisions are highlighted in yellow font in the revised manuscript. Our suggested revisions follow:

Authors Response to PLOS ONE Reviewers.
We thank the reviewers for useful suggestions to improve and revise the manuscript.

Manuscript Review: Reviewer's Responses to Questions.

Methods:

Reviewer # 1. No author response required.

Reviewer # 2. A clarification of the purpose, framework, and components for the proposed Place-Based Conceptual Model has been added to the text and in a revised Figure 6. Some examples of how the PBCM could be used as a template to organize and compare before and after dam habitat descriptions have been added to the text.

Reviewer # 3. Provided an accurate description of the manuscripts purpose and content.

Reviewer # 4. A revised description with details of the objectives of the study has been added as requested and the text has been edited for clarification. A revised Fig. 6 was added to clarify the revised study objectives. Examples of how the PBCM could be used to aggregate and compare habitat descriptions before and after dam construction have been added to the text.

Results/Discussion/Conclusions:

Reviewer # 1. No author response required.

Reviewer # 2. As suggested, an example of a maxent model describing snail habitat as compared to the PBCM in a generally similar context has been added to the revised text beginning with line 391.

Reviewer # 3. No author response required.

Reviewer # 4. The manuscript has been completely reorganized and rewritten with responses to the points raised in Methods above. Information has added to the text to clarify the study discussion. Beginning with line 152 of the revised manuscript, the PBCM has been integrated throughout the manuscript as suggested using selected review/modeling scenarios that are compatible with descriptions from the PBCM.

<u>Recommend "Minor Revision" or "Accept"/Summary and General Comments:</u>

Reviewer # 1. All requested changes have been made in the text. Text has been added beginning at Line 113 of the revised text describing potential changes in naturally occurring fish and turtle predators of snails with changing conditions.

Reviewer # 2. No response received.

Reviewer # 3. All minor comments have been addressed in the text.

Reviewer # 4. Lines 6 -72 and 111-131 have been moved to the Introduction as suggested and a clarification of the purpose of the proposed PBCM framework have been added to the Introduction at lines 23-30 of the revised text. The text has been focused and edited as requested.

The Title and Abstract have been revised to reflect the requested revisions in the manuscript. A completely revised Figure 6 Place-based Conceptual Model (PBCM) of *Neotricula aperta/Schistosoma mekongi* Habitat in the Mekong River has been created in TIFF format.

Guy R. Lanza
E. Suchart Upatham
Ang Chen

Click here to access/download
Revised Article with Changes Highlighted
PLOS Y.docx
