# Supplementary material for: A Place-Based Conceptual Model (PBCM) of Neotricula aperta/Schistosoma mekongi habitat before and after dam construction in the Lower Mekong River
Source: PLoS Negl Trop Dis. 2023 Oct 6;17(10):e0011122. doi: 10.1371/journal.pntd.0011122 (PMC10584140; doi:10.1371/journal.pntd.0011122)
Supplement: Attachment — Submitted filename: Response to PLOS ONE Reviewers.docx [file pntd.0011122.s003.docx]

April 9, 2023

Professor Krystyna Cwiklinski

Academic Editor

PLOS Neglected Tropical Diseases

Professor Alvaro Acosta-Serrano

Section Editor,

PLOS Neglected Tropical Diseases

Dear Professors Cwiklinski and Acosta-Serrano,

We have revised PNTD-D-23-00131(1)jrs.pdf using the helpful suggestions of the reviewers to clarify and improve the text. The revisions are highlighted in yellow font in the revised manuscript. Our suggested revisions follow:

Authors Response to PLOS ONE Reviewers.

We thank the reviewers for useful suggestions to improve and revise the manuscript.

**Manuscript Review: Reviewer’s Responses to Questions.**

***Methods:***

Reviewer # 1. No author response required.

Reviewer # 2. A clarification of the purpose, framework, and components for the proposed Place-Based Conceptual Model has been added to the text and in a revised Figure 6. Some examples of how the PBCM could be used as a template to organize and compare before and after dam habitat descriptions have been added to the text.

Reviewer # 3. Provided an accurate description of the manuscripts purpose and content.

Reviewer # 4. A revised description with details of the objectives of the study has been added as requested and the text has been edited for clarification. A revised Fig. 6 was added to clarify the revised study objectives. Examples of how the PBCM could be used to aggregate and compare habitat descriptions before and after dam construction have been added to the text.

***Results/Discussion/Conclusions:***

Reviewer # 1. No author response required.

Reviewer # 2. As suggested, an example of a maxent model describing snail habitat as compared to the PBCM in a generally similar context has been added to the revised text beginning with line 391.

Reviewer # 3. No author response required.

Reviewer # 4. The manuscript has been completely reorganized and rewritten with responses to the points raised in Methods above. Information has added to the text to clarify the study discussion. Beginning with line 152 of the revised manuscript, the PBCM has been integrated throughout the manuscript as suggested using selected review/modeling scenarios that are compatible with descriptions from the PBCM.

***Recommend “Minor Revision” or “Accept”/Summary and General Comments:***

Reviewer # 1. All requested changes have been made in the text. Text has been added beginning at Line 113 of the revised text describing potential changes in naturally occurring fish and turtle predators of snails with changing conditions.

Reviewer # 2. No response received.

Reviewer # 3. All minor comments have been addressed in the text.

Reviewer # 4. Lines 6 -72 and 111-131 have been moved to the Introduction as suggested and a clarification of the purpose of the proposed PBCM framework have been added to the Introduction at lines 23-30 of the revised text. The text has been focused and edited as requested.

The Title and Abstract have been revised to reflect the requested revisions in the manuscript.

A completely revised Figure 6 Place-based Conceptual Model (PBCM) of *Neotricula aperta/Schistosoma mekongi* Habitat in the Mekong River has been created in TIFF format.

Guy R. Lanza

E. Suchart Upatham

Ang Chen
