# Supplementary material for: A Place-Based Conceptual Model (PBCM) of Neotricula aperta/Schistosoma mekongi habitat before and after dam construction in the Lower Mekong River
Source: PLoS Negl Trop Dis. 2023 Oct 6;17(10):e0011122. doi: 10.1371/journal.pntd.0011122 (PMC10584140; doi:10.1371/journal.pntd.0011122)
Supplement: Attachment — Submitted filename: Response to PLOS ONE Reviewers copy.docx [file pntd.0011122.s005.docx]

August 25, 2023

Professor Krystyna Cwiklinski

Academic Editor

PLOS Neglected Tropical Diseases

Professor Alvaro Acosta-Serrano

Section Editor,

PLOS Neglected Tropical Diseases

Dear Professors Cwiklinski and Acosta-Serrano,

We have completely revised/rewritten PNTD-D-23-00131(1)jrs.pdf using the helpful suggestions of the reviewers to clarify and improve the text. We include the current revised version (clean copy) and the version with the changes highlighted in yellow as per your request. All numerical data used to create Figures 4-7 are in the text and a data file of numerical values used for Figure 8 is attached as a supplement. Our detailed revisions follow:

Authors Response to PLOS ONE Reviewers.

We thank the reviewers for useful suggestions to improve and revise the manuscript.

All requested revisions have been made in the title, on Lines 5, 24, 55, 73-74, 80-82, 114-115, 165, 199, 203, 210, 213, 215, 394, 444-445, 447,

Guy R. Lanza

Suchart Upatham

Ang Chen
